# Memory-dictated dynamics of single-atom Pt on CeO_2_ for CO oxidation

**DOI:** 10.1038/s41467-023-37776-3

**Published:** 2023-05-09

**Authors:** Zihao Zhang, Jinshu Tian, Yubing Lu, Shize Yang, Dong Jiang, Weixin Huang, Yixiao Li, Jiyun Hong, Adam S. Hoffman, Simon R. Bare, Mark H. Engelhard, Abhaya K. Datye, Yong Wang

**Affiliations:** 1 Institute for Integrated Catalysis, Pacific Northwest National Laboratory, Richland, WA 99354 USA; 2 The Gene and Linda Voiland School of Chemical Engineering and Bioengineering, Washington State University, Pullman, WA 99164 USA; 3 Eyring Materials Center, Arizona State University, Tempe, AZ 85257 USA; 4 Stanford Synchrotron Radiation Light Source, SLAC National Accelerator Laboratory, Menlo Park, CA 94025 USA; 5 Department of Chemical and Biological Engineering and Center for Micro-Engineered Materials, University of New Mexico, Albuquerque, NM 87131 USA

**Keywords:** Chemical engineering, Heterogeneous catalysis

## Abstract

Single atoms of platinum group metals on CeO_2_ represent a potential approach to lower precious metal requirements for automobile exhaust treatment catalysts. Here we show the dynamic evolution of two types of single-atom Pt (Pt_1_) on CeO_2_, i.e., adsorbed Pt_1_ in Pt/CeO_2_ and square planar Pt_1_ in Pt_AT_CeO_2_, fabricated at 500 °C and by atom-trapping method at 800 °C, respectively. Adsorbed Pt_1_ in Pt/CeO_2_ is mobile with the in situ formation of few-atom Pt clusters during CO oxidation, contributing to high reactivity with near-zero reaction order in CO. In contrast, square planar Pt_1_ in Pt_AT_CeO_2_ is strongly anchored to the support during CO oxidation leading to relatively low reactivity with a positive reaction order in CO. Reduction of both Pt/CeO_2_ and Pt_AT_CeO_2_ in CO transforms Pt_1_ to Pt nanoparticles. However, both catalysts retain the memory of their initial Pt_1_ state after reoxidative treatments, which illustrates the importance of the initial single-atom structure in practical applications.

## Abstract (web summary)

The use of single atoms of platinum group metals on CeO_2_ is a promising approach to reduce precious metal requirements for automobile exhaust treatment catalysts. Here, the authors discovered that by manipulating the calcination temperatures, they could control the configuration of Pt1 on the CeO_2_ surface, leading to differences in CO oxidation activity.

## Introduction

Single-atom catalysts (SACs) have been attracting widespread attention in the catalysis community for the past decade^1,2^. Among them, CeO_2_-supported SACs are particularly interesting because of the oxygen storage capacity of CeO_2_ and the ability of CeO_2_ to intrinsically trap platinum group metals (PGMs: Pt, Pd, Rh, etc.) under high-temperature oxidative condition^3–6^. CeO_2_-supported PGMs prepared by atom-trapping (AT) method at 800 °C have recently been reported to be promising sintering-resistant catalysts for the removal of vehicle criteria pollutants (e.g., CO, NO_x_, and hydrocarbons)^7–9^. While the maximum atomic utilization can be realized for isolated PGMs (e.g., Pt_1_), the intrinsic activity of Pt_1_ is usually lower than Pt aggregates^10–12^. To circumvent this issue, Pt_1_ on CeO_2_ was transformed to more active Pt nanoparticles (NPs, <2 nm) via the treatment in reducing atmospheres (CO, H_2_, or HCs) at elevated temperatures^10,13,14^. However, these agglomerated Pt NPs redisperse into less-active Pt_1_ under an additional treatment in O_2_ or even in a lean reaction condition at temperatures higher than 400 °C^15,16^, which complicates their applications in practical exhaust gas treatment.

**Fig. 1:**
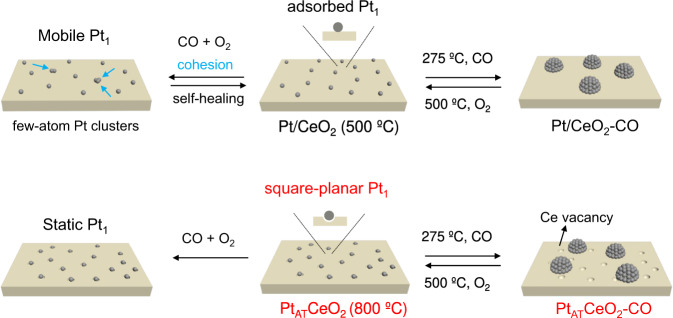
Dynamics of Pt_1_ under different conditions. Schematic illustration of dynamic behaviors of single-atom Pt_1_ in Pt/CeO_2_ and Pt_AT_CeO_2_ under CO oxidation, reductive, and oxidative conditions at elevated temperatures.

It has been reported that single-atom Pt, Pd, or Cu on CeO_2_ fabricated by different annealing temperatures show various catalytic performances^13,17,18^. However, the origin of significant reactivity difference induced by different annealing temperatures is still unknown^4,7–10^. Although the dynamics of Pt_1_ under reductive and oxidative conditions are both studied, the dynamic evolution of different types of Pt_1_ under a real reaction condition is still missing. Understanding the initial Pt_1_ structure and its dynamics under reaction conditions is of great importance to design more efficient Pt_1_ or its derived active site for practical exhaust gas treatment. Therefore, two types of Pt_1_ on CeO_2_ catalysts were fabricated, one via treatment at 500 °C (Pt/CeO_2_) and the second by atom-trapping method at 800 °C (Pt_AT_CeO_2_). The local structure and dynamic behavior of the two Pt_1_ structures under CO oxidation condition were studied by in situ X-ray absorption spectroscopy (XAS), in situ infrared spectroscopy, quasi in situ X-ray photoelectron spectroscopy (XPS), and density functional theory (DFT) calculations. Both types of Pt_1_ structures were studied under treatment in CO at 275 °C which led to the formation of Pt NPs in both Pt/CeO_2_-CO and Pt_AT_CeO_2_-CO, followed by a reoxidative treatment at 500 °C to disintegrate the as-formed Pt NPs to form Pt_1_ again in Pt/CeO_2_-CO-O_2_ and Pt_AT_CeO_2_-CO-O_2_ (Fig. 1). The dynamics of Pt_1_ under CO oxidation, reductive and oxidative treatments are investigated by comparing their CO oxidation activity, reaction kinetics, characterization results, and theoretical calculations.

## Results and discussion

### Single-atom Pt_1_ structure in fresh Pt/CeO_2_ and Pt_AT_CeO_2_

**Fig. 2:**
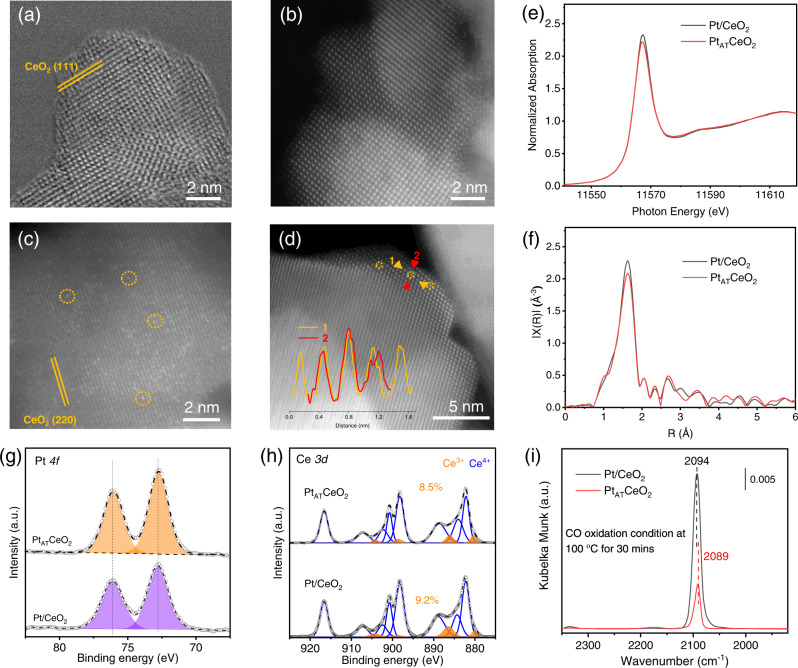
Ex situ characterizations. HAADF-STEM images of **a**, **b** Pt/CeO_2_ and **c**, **d** Pt_AT_CeO_2_ (Pt_1_ is marked as cycles, and line-scanning of a single Pt_1_ is shown in the inset of (**d**). **e** Pt *L*_*3*_-edge XANES and **f** the corresponding magnitude of the Fourier transform of the EXAFS spectra of Pt/CeO_2_ and Pt_AT_CeO_2_. **g**, **h** Pt *4f* and Ce *3d* XPS spectra of Pt/CeO_2_ and Pt_AT_CeO_2_. **i** In situ CO diffuse-reflectance infrared Fourier transform spectra (CO-DRIFTS) for Pt/CeO_2_ and Pt_AT_CeO_2_ under CO oxidation condition at 100 °C for 30 min.

Pt/CeO_2_ and Pt_AT_CeO_2_ with ~1 wt% Pt loading (Supplementary Table S1) were synthesized by two post-calcination temperatures of 500 and 800 °C in air, in which the calcination temperature of 800 °C represents a previously reported atom-trapping method^8^. Aberration-corrected high-angle annular dark-field scanning transmission electron microscopy (HAADF-STEM) images in Fig. 2a–d and Supplementary Fig. S1, and line-scanning results in the inset of Fig. 2d display that isolated Pt_1_ atoms are present in Pt/CeO_2_ and Pt_AT_CeO_2_. The powder X-ray diffraction (XRD) patterns of Pt/CeO_2_ and Pt_AT_CeO_2_ in Supplementary Fig. S2 show only the diffraction peaks for fluorite CeO_2_. Pt *L*_*3*_-edge X-ray absorption near edge structure (XANES) spectroscopy in Fig. 2e, Supplementary Fig. S3 exhibits a white line intensity slightly lower than the PtO_2_ (Pt^4+^) reference, indicating a cationic Pt^σ+^ nature (σ < 4)^19^. In contrast, Pt *4f* X-ray photoelectron spectroscopy (XPS) in Fig. 2g displays a similar characteristic of Pt^2+^ for two fresh samples^3^. The observed different Pt valences (Pt^2+^ in XPS, near Pt^4+^ in XANES) are mainly ascribed to various oxygen partial pressures in XANES (ambient air) and XPS (vacuum) measurement conditions^20^. Both the percentage of surface Ce^3+^ and the defect-related O in Pt/CeO_2_ and Pt_AT_CeO_2_ are similar (Fig. 2h, Supplementary Fig. S4). The extended X-ray absorption fine structure (EXAFS) results in Fig. 2f, Supplementary Fig. S3, display that the two samples are dominated by the first-shell Pt-O contribution, and the corresponding coordination number (CN) is 5.0 ± 0.43 for Pt/CeO_2_, and 4.9 ± 0.52 for Pt_AT_CeO_2_ (Supplementary Table S2, Supplementary Figs. S5 and S6). The above ex situ characterizations suggest that the two fresh catalysts have the same atomically dispersed nature, similar Pt valence, similar Pt-O local coordination, and similar Ce^3+^ and defect-related O percentage. However, their difference can be revealed by diffuse-reflectance infrared Fourier transform spectra with CO as a probe molecule (CO-DRIFTS) (Fig. 2i, Supplementary Fig. S7), which reveals a single IR band at ~2094 for Pt/CeO_2_ and ~2089 cm^−1^ for Pt_AT_CeO_2_ at 100 °C under CO oxidation condition, ascribed to CO linearly adsorbed on ionic Pt^21^. The different vibration frequencies of adsorbed CO molecules can be tentatively assigned to their different CO-Pt_1_ interactions under CO oxidation condition^22^. This implies the possible structural change of Pt_1_ from ambient air to CO oxidation condition for Pt/CeO_2_ or Pt_AT_CeO_2_. Previous studies reported that Pt_1_ on CeO_2_ synthesized by atom-trapping method holds a square planar structure^7,21,23^; however, Pt_1_ structure synthesized at low calcination temperature is less discussed.

### CO oxidation activity and reaction kinetics

**Fig. 3:**
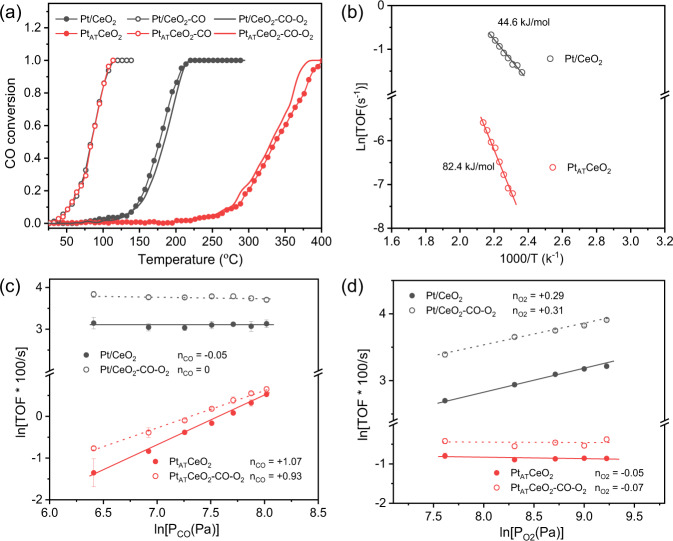
Catalytic evaluation. **a** CO oxidation performance (light-off curve) with 20 mg catalyst, and **b** Arrhenius plots of Pt/CeO_2_ and Pt_AT_CeO_2_ with different catalyst loadings (4 mg Pt/CeO_2_, 300 mg Pt_AT_CeO_2_). Reaction conditions: 1% CO and 4% O_2_ balanced with Ar, catalyst diluted with SiC to 400 mg, total flow rate = 100 mL/min. Effect of **c** CO and **d** O_2_ partial pressure on the reaction rate (TOF). Measurement conditions: P_CO_ = 0.6–3 kPa, P_O2_ = 4 kPa in (**c**); P_CO_ = 1 kPa, P_O2_ = 2–10 kPa in (**d**). The operating temperatures for Pt/CeO_2_, Pt_AT_CeO_2_, Pt/CeO_2_-CO-O_2_, and Pt_AT_CeO_2_-CO-O_2_ are 210, 210, 220, and 215 °C, respectively, in (**c**, **d**).

Pt/CeO_2_ and Pt_AT_CeO_2_ were then evaluated for CO oxidation under O_2_-rich (lean) conditions with a weight hourly space velocity (WHSV) of 300 L/g*h. The light-off curves and corresponding Arrhenius plots in Fig. 3a and Supplementary Fig. S8 show that Pt/CeO_2_ is more active than Pt_AT_CeO_2_. For instance, T_50_ (temperature for 50% CO conversion) for Pt/CeO_2_ and Pt_AT_CeO_2_ are 180 and 335 °C, respectively. Five repeated light-off curves (Supplementary Fig. S9) display that two catalysts show stable catalytic performance and are both more active than the pristine CeO_2_ (Supplementary Fig. S10). The obtained apparent activation energies (E_a_) of Pt/CeO_2_ and Pt_AT_CeO_2_ in the same temperature region (160–215 °C) by changing the WHSV are 44.6 and 82.4 kJ/mol (Fig. 3b), suggesting the reaction energy barrier in Pt/CeO_2_ is distinctly lower than that in Pt_AT_CeO_2_. Moreover, the reaction order at ~200 °C in CO is ~0 for Pt/CeO_2_ but +1 for Pt_AT_CeO_2_ (Fig. 3c). The near-zero reaction order in CO suggests the more favorable CO adsorption on Pt/CeO_2_, which can be confirmed by a higher intensity of adsorbed CO peak in CO-DRIFTS (Fig. 2i) and more CO_2_ evolution in temperature-programmed desorption of CO (CO-TPD, Supplementary Fig. S11). The kinetic feature of Pt/CeO_2_ is also similar to that of reduced Pt/CeO_2_ and Pt_AT_CeO_2_ samples obtained after a reduction in CO at 275 °C (Supplementary Fig. S12), as well as the Pt or Pd clusters on CeO_2_^11,24^. This indicates that Pt_1_ might sinter in Pt/CeO_2_ under CO oxidation conditions. Increasing the surface coverage of Pt_1_ is observed for two SACs with increasing CO partial pressure in CO-DRIFTS experiments at 100 °C (Supplementary Fig. S13a, b). However, the surface CO coverage in Pt/CeO_2_ is higher than that in Pt_AT_CeO_2_ under the same condition, suggesting CO adsorption on Pt/CeO_2_ is more kinetic-irrelevant, in agreement with the results in Fig. 3c. Moreover, the reaction orders in O_2_ (Fig. 3d) over the two catalysts are also different, i.e., +0.3 for Pt/CeO_2_ and ~0 for Pt_AT_CeO_2_. Based on O_2_-dependent CO-DRIFTS results (Supplementary Fig. S13c, d), higher O_2_ partial pressure leads to a higher surface CO coverage on Pt/CeO_2_; however, O_2_ partial pressure does not have a noticeable effect on CO coverage on Pt_AT_CeO_2_. The above activity and kinetics suggest different dynamic behaviors of Pt_1_ in Pt/CeO_2_ and Pt_AT_CeO_2_ under CO oxidation condition. Both CO-DRIFTS (Fig. 2i) and CO oxidation kinetics suggest that two SACs hold different Pt_1_ structures under CO oxidation condition.

Additionally, Pt/CeO_2_ and Pt_AT_CeO_2_ with a lower Pt loading (~0.1 wt%) also display a similar activity trend (Supplementary Figs. S14–S18). The detailed discussion is provided in Supplementary Information after Supplementary Figs. S14 and S15. It has been reported that surface reconstruction of CeO_2_ at different calcination temperatures would affect the catalytic activity^25^. To minimize these effects, the CeO_2_ support was pre-calcined at 800 °C for 10 h to yield 800CeO_2_, followed by deposition of 0.1 wt% Pt (to maintain the atomically dispersed nature) at 500 and 800 °C to yield 0.1Pt/800CeO_2_ and 0.1Pt_AT_800CeO_2_, respectively. Since the support was pre-calcined at 800 °C, these two samples exhibited similar porosity properties (Supplementary Fig. S17, Supplementary Table S3) and CeO_2_ particle size (Supplementary Fig. S18) as the 800CeO_2_ support. We found that the activity of 0.1Pt/800CeO_2_ was still significantly higher than that of 0.1Pt_AT_800CeO_2_ (Supplementary Fig. S14), similar to Pt/CeO_2_ and Pt_AT_CeO_2_ (Fig. 3a).

### Dynamic evolution under CO oxidation condition

**Fig. 4:**
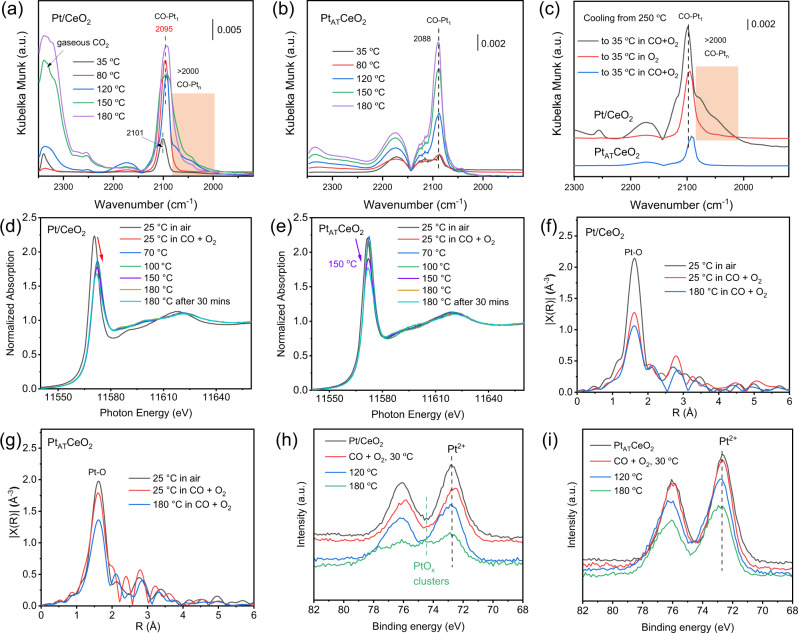
In situ characterizations. In situ CO-DRIFTS for **a** Pt/CeO_2_ and **b** Pt_AT_CeO_2_ in CO and O_2_ mixture as varying the temperature from 35 to 180 °C, as well as **c** the CO-DRIFTS after reaction at 250 °C, and cooling down to 35 °C in CO + O_2_ or O_2_. Pt *L*_*3*_-edge in situ XANES of **d** Pt/CeO_2_, and **e** Pt_AT_CeO_2_ at 25 °C in ambient air and different reaction temperatures (25, 70, 100, 150, 180 °C) in CO oxidation condition (CO/O_2_ ratio is 1:4). the corresponding magnitude of the Fourier transform of the EXAFS of **f** Pt/CeO_2_ and **g** Pt_AT_CeO_2_ at 25 °C in ambient air, and at 25 °C, 180 °C under reaction condition, k = 3–12.5 Å^−1^ for the Fourier transform. Quasi in situ Pt *4f* XPS spectra for **h** Pt/CeO_2_ and **i** Pt_AT_CeO_2_ without or with treatment at different reaction temperatures (CO/O_2_ ratio is 1:4) for 20 min. After treatment, gases were pumped for the XPS test.

To probe the activity origin of Pt/CeO_2_ and Pt_AT_CeO_2_, in situ CO-DRIFTS was first performed under CO oxidation conditions at different temperatures. At 35 or 80 °C, Pt_AT_CeO_2_ shows a similar weak IR peak centered at ~2088 cm^−1^ (Fig. 4b)^12,19^, which becomes increasingly apparent at 120 °C. It has been reported that square planar Pt_1_ hardly chemisorbs CO^26,27^, and high-temperature treatment in CO + O_2_ reconstructs new Pt_1_ cations, which are capable of adsorbing CO^22^, in agreement with our Pt_AT_CeO_2_ results. In contrast, Pt/CeO_2_ shows a stronger adsorbed CO-Pt_1_ peak (~2101 cm^−1^) at 35 °C (Fig. 4a), which becomes more intense at 80 °C with a red shift, suggesting a possible reduction of Pt_1_. An obvious shoulder (2000–2060 cm^−1^) is observed in Pt/CeO_2_ above 120 °C, along with an increased intensity of gaseous CO_2_ in the IR cell, indicating that the reaction has begun and a new Pt species has formed. This shoulder is a typical characteristic of Pt clusters^10,11^. The above results show the reduction and sintering of Pt_1_ in Pt/CeO_2_ under the elevated reaction temperature. However, the features (<2000 cm^−1^) ascribed to bridge adsorbed CO on the traditional large Pt NPs^28–30^ are not observed, which can be seen in the reduced Pt/CeO_2_ and Pt_AT_CeO_2_ (Supplementary Fig. S19). After cooling down to 35 °C in CO + O_2_ from 250 °C, the Pt clusters feature can still be found (Fig. 4c). This feature disappears after cooling down in O_2_, suggesting as-formed Pt clusters completely redisperse on CeO_2_ in O_2_. These suggest that Pt_1_ in Pt/CeO_2_ may only sinter into few-atom Pt clusters under reaction conditions due to the presence of self-healing of Pt clusters into Pt_1_ under O_2_-rich condition. The co-existence of Pt_1_ cohesion and self-healing of Pt clusters in Pt/CeO_2_ is crucial to maintain fully exposed Pt clusters under CO oxidation condition^31^. It should be emphasized that a gaseous CO_2_ signal shows up at 35 °C and then disappears at 80 °C for Pt/CeO_2_ in CO-DRIFTS (Fig. 4a). To validate this phenomenon, we then performed the temperature-programmed surface reaction (Supplementary Fig. S20). Once CO is introduced in O_2_-treated samples at 35 °C, an immediate and ephemeral CO_2_ evolution together with CO consumption is found only in Pt/CeO_2_, suggesting the active O (or weakly bonded O) in Pt/CeO_2_ can react with CO to form CO_2_ at 35 °C. Meanwhile, a surface Pt_1_ reconstruction in Pt/CeO_2_ must occur due to the loss of surface O.

To further study the dynamic evolution of Pt_1_, in situ XANES data were collected under CO oxidation condition. For Pt/CeO_2_, an obvious decrease of the white line intensity is observed while switching the exposed atmosphere from ambient air to CO and O_2_ at 25 °C (Fig. 4d), indicating Pt_1_ transforms from near Pt^4+^ in ambient air to ~Pt^2+^ in CO and O_2_, as compared with the Pt reference (Supplementary Fig. S21). This finding explains why a CO_2_ signal is observed at 35 °C in Pt/CeO_2_ after introducing CO + O_2_ (Fig. 4a, Supplementary Fig. S20). The decreased Pt valence can also be evidenced by the decreased first-shell Pt-O CN (5 to 3.1) from in situ EXAFS (Fig. 4f, Supplementary Table S2), clearly indicating the abovementioned active O in Pt/CeO_2_ directly bonds with Pt_1_. As further increasing temperature to 180 °C in CO + O_2_, Pt valence in Pt/CeO_2_ descends slowly (Fig. 4d). In contrast, white line intensity in Pt_AT_CeO_2_ is stable after flowing CO and O_2_ at 25 °C or even at 100 °C, and it decreases only at 150 °C (Fig. 4e). The Pt-O CN decreases from 4.9 at 25 °C to 3.2 at 180 °C (Fig. 4g), indicating Pt_1_ in Pt_AT_CeO_2_ reconstructs into lower-valence Pt_1_ at increased temperature. Nonetheless, Pt-O CN in Pt_AT_CeO_2_ at 180 °C is still higher than 2.8 found in Pt/CeO_2_ (Supplementary Table S2). After CO oxidation treatment at different temperatures, XPS data were collected quasi in situ. For Pt/CeO_2_, the mild treatment at 30 °C does not influence the XPS signal, but a new feature appears at 180 °C, as seen in Fig. 4h. This suggests the formation of Pt species with the valence higher than 2. Based on the previous studies^30^, the oxidation of Pt NPs to PtO_2_/PtO cluster mixture or the formation of thin PtO_x_ oxide film can induce the formation of Pt cations (>2+). In comparison, this new Pt feature is not observed in Pt_AT_CeO_2_ under the same treatment condition (Fig. 4i). Therefore, we ascribe the newly formed Pt species under CO oxidation condition in Pt/CeO_2_ as few-atom Pt clusters (Fig. 1). Moreover, the relatively lower Pt-O CN in Pt/CeO_2_ at 180 °C is ascribed to the formation of few-atom Pt clusters under reaction condition by combining with CO-DRIFTS, XPS, and kinetics studies.

### Dynamic evolution under reductive-oxidative cycle and structural memory

**Fig. 5:**
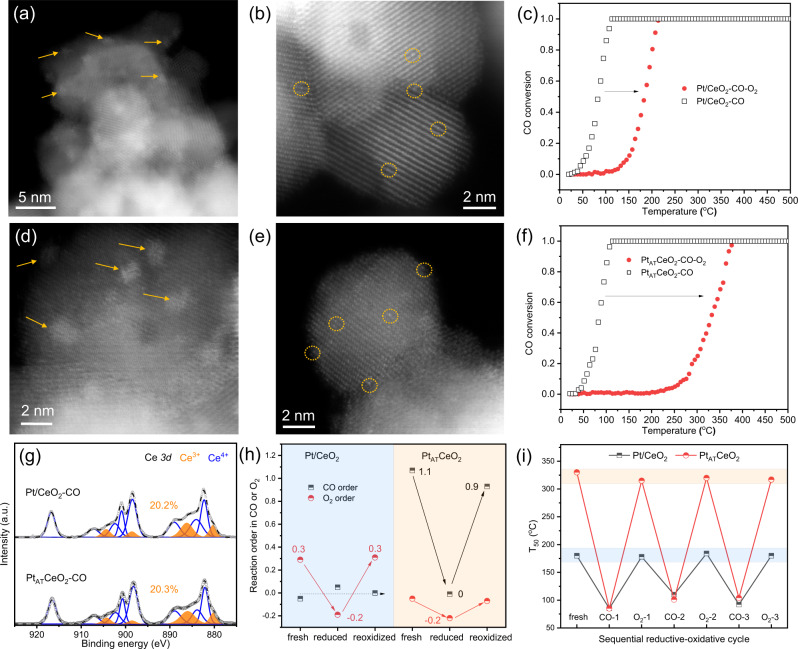
Structural memory under reductive-oxidative cycle. STEM images of **a** Pt/CeO_2_-CO, **b** Pt/CeO_2_-CO-O_2_, **d** Pt_AT_CeO_2_-CO, and **e** Pt_AT_CeO_2_-CO-O_2_. Light-off curves of reduced and reoxidized **c** Pt/CeO_2_ and **f** Pt_AT_CeO_2_. **g** Ce *3d* XPS spectra of Pt/CeO_2_-CO and Pt_AT_CeO_2_-CO; XPS data are collected after treatment without exposure to air. **h** Reaction orders in CO and O_2_ for Pt/CeO_2_ and Pt_AT_CeO_2_ in three states. **i** T_50_ results of Pt/CeO_2_ and Pt_AT_CeO_2_ after a sequential reductive-oxidative cycle. Reaction conditions in (**c**, **f**, **i**) are the same as that in Fig. 3a.

To further investigate the difference between the two Pt_1_ configurations, we designed a cohesion-redispersion cycle experiment. Two SACs are first treated in CO at 275 °C to form Pt/CeO_2_-CO and Pt_AT_CeO_2_-CO. Pt NPs (1–2 nm in size) in reduced samples can be evidenced by HAADF-STEM images (Fig. 5a, d, Supplementary Fig. S22), XPS^32–34^ (Supplementary Fig. S23), CO-DRIFTS (Supplementary Fig. S19), and Raman spectroscopy (Supplementary Fig. S24). Pt/CeO_2_-CO and Pt_AT_CeO_2_-CO show similar enhanced CO oxidation reactivity (Fig. 3a) and similar reaction orders (Supplementary Fig. S12), ascribed to the presence of Pt clusters^13^. The percentage of Ce^3+^ and surface defect-related O also increases after CO reduction (Fig. 5g, Supplementary Fig. S25). However, the increased activity is lost during the repeated CO oxidation experiments from 25 to 500 °C for both reduced catalysts (Supplementary Fig. S26). If we treat Pt/CeO_2_-CO and Pt_AT_CeO_2_-CO in O_2_ at 500 °C, both enhanced activities will also decrease and become similar to their respective initial activity (Fig. 5c, f). The activity loss is due to the redispersion of Pt NPs into Pt_1_ evidenced by HAADF-STEM images (Fig. 5b, e, Supplementary Fig. S27) and CO-DRIFTS (Supplementary Figs. S28 and S29) results. Moreover, adsorbed CO-Pt_1_ peak (Supplementary Figs. S28 and S29) in reoxidized Pt/CeO_2_-CO-O_2_ and Pt_AT_CeO_2_-CO-O_2_ is located at ~2095 and ~2089 cm^−1^, respectively, that is consistent with their respective fresh sample (Fig. 2i). This implies that two kinds of Pt_1_ appear to have memory back to their initial state after a cohesion-redispersion cycle. More interestingly, the reaction kinetics also shows a similar memory behavior. Specifically, the reaction orders in CO for Pt/CeO_2_ in three states (fresh-reduced-reoxidized) are all closer to 0 but change from 0.3 through −0.2 to 0.3 in O_2_ (Fig. 5h). In Pt_AT_CeO_2_, the reaction order changes from 1.1 through 0 to 0.9 in CO, and from 0 through −0.2 to 0 in O_2_. We also reduced Pt_1_ in Pt/CeO_2_ and Pt_AT_CeO_2_ with H_2_ instead of CO, and the enhanced reactivity was also lost after a further reoxidation treatment at 500 °C (Supplementary Fig. S30). This indicates two catalysts have structural memory after both CO-O_2_ and H_2_-O_2_ treatment cycles. Furthermore, T_50_ of Pt/CeO_2_ and Pt_AT_CeO_2_ after the sequential reductive-oxidative cycle (Fig. 5i) show that the cohesion-redispersion behavior of Pt_1_ can be repeated many times. Therefore, we believe that after a reduction-reoxidation cycle, two Pt_1_ configurations in Pt/CeO_2_ and Pt_AT_CeO_2_ both return to their initial structure.

### Theoretical insight into the dynamic behaviors

**Fig. 6:**
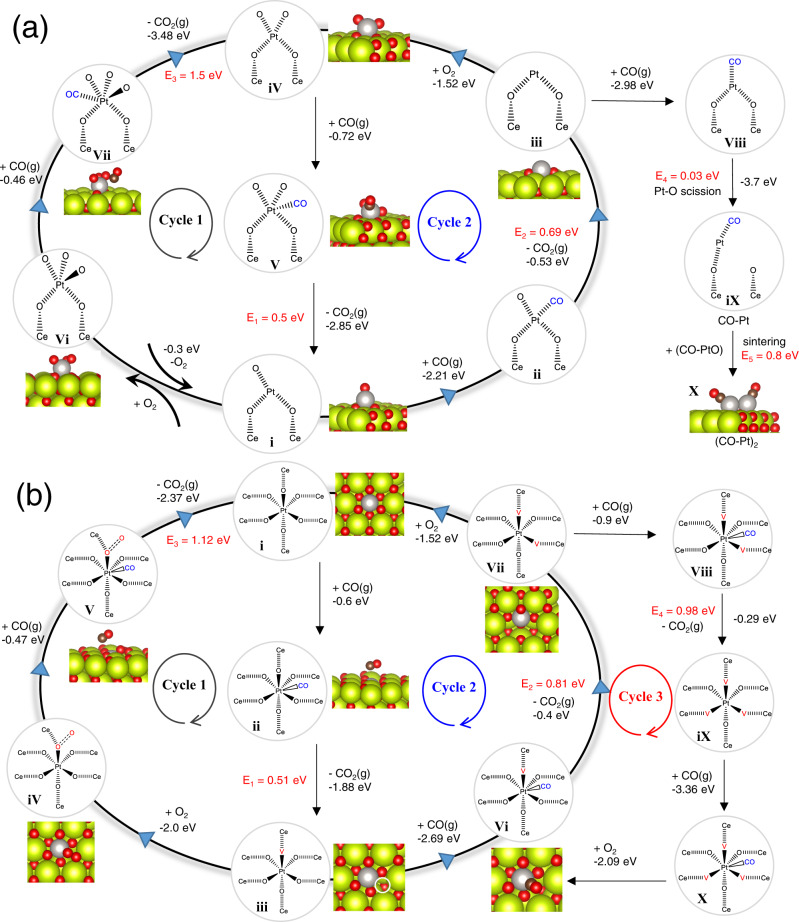
DFT calculation. CO oxidation reaction mechanism on **a** adsorbed Pt_1_ and **b** square planar Pt_1_ on CeO_2_ (111) terrace. The main model structures are shown in the reaction cycle.

To explain the above dynamic behaviors, the nascent Pt_1_ structures of Pt/CeO_2_ and Pt_AT_CeO_2_ are identified first. Based on the previous studies^7,21–23^, Pt_AT_CeO_2_ is dominated by square planar Pt_1_ structure on CeO_2_(111) terrace (Supplementary Fig. S31b) or step site (Supplementary Fig. S31c). However, Pt_1_ configuration in Pt/CeO_2_ is still unknown. To understand if Pt_1_ in Pt/CeO_2_ is another reported single-atom structure–adsorbed Pt_1_ (Supplementary Fig. S31a)^35,36^, we first compare EXAFS fitting results of Pt/CeO_2_ and adsorbed Pt_1_ models (Supplementary Fig. S31d), and the adsorbed PtO_5_ model on CeO_2_ (111) fits well with Pt/CeO_2_. Then, we calculate the oxygen vacancy (V_O_) formation energy of neighboring O of both adsorbed Pt_1_ and square planar Pt_1_. It is found that the V_O_ formation energy of adsorbed Pt_1_ is significantly lower than that of square planar Pt_1_ (Supplementary Fig. S32). This indicates that neighboring O atoms of adsorbed Pt_1_ are easier to remove, consistent with previous results (Fig. 4a, d, Supplementary Fig. S20), which further justifies our proposed adsorbed Pt_1_ model for Pt/CeO_2_. Therefore, we assume that our Pt/CeO_2_ is mainly composed of adsorbed PtO_5_ structure in air (Supplementary Fig. S31a). Under CO oxidation, the adsorbed PtO_5_ adsorbs CO with the adsorption energy of −0.46 eV (Vi to Vii, Fig. 6a), but the adsorbed CO-PtO_5_ is difficult to release CO_2_ with an energy barrier of 1.5 eV (Vii to iV). Therefore, cycle 1 in Fig. 6a is unlikely to occur. Instead, the PtO_5_ structure can easily transform into a PtO_3_ structure (Vi to i) with an exothermic energy of −0.3 eV, consistent with in situ XAS result (Fig. 4d). The formed PtO_3_ adsorbs CO strongly (i to ii), then releases CO_2_ to form PtO_2_ with an energy barrier of 0.69 eV (ii to iii). O_2_ fills the O_v_ around PtO_2_ to form PtO_4_ with an exothermic energy of −1.52 eV, followed by a CO adsorption (iii to iV to V). The adsorbed CO-PtO_4_ loses CO_2_ to form PtO_3_ with the energy barrier of 0.5 eV to complete cycle 2. PtO_2_ can also adsorb CO strongly to form CO-PtO_2_; however, Pt-O scission occurs spontaneously with a strong exothermic energy of −3.7 eV (Viii to iX) to form CO-PtO structure (iX). Assuming that there are two CO-PtO on the CeO_2_ surface, the calculated Pt-Pt cohesion energy barrier is 0.8 eV (iX to X), which indicates the possible Pt-Pt cohesion under reaction condition in Pt/CeO_2_.

In Fig. 6b, square planar Pt_1_ on CeO_2_(111) terrace is adpoted^23^. First, CO adsorbs on square planar Pt_1_ (PtO_6_) with an adsorption energy of −0.6 eV (i to ii), much lower than −2.21 eV observed on PtO_3_ in Pt/CeO_2_. This is consistent with the stronger IR signal found in Pt/CeO_2_ at 35 and 80 °C (Fig. 4a, b). Thereafter, CO-PtO_6_ requires a moderate energy barrier of 0.5 eV to release CO_2_ to form PtO_5_ (ii to iii), which is why the white line intensity of Pt_AT_CeO_2_ only decreases at 150 °C (Fig. 4e). PtO_5_ can either adsorb O_2_ or CO to form PtO_5_(O_2_) (iii to iV) or CO-PtO_5_ (iii to Vi). However, CO adsorbed on PtO_5_(O_2_) requires an energy barrier of 1.12 eV to release CO_2_ (V to i), which makes cycle 1 less favorable. In contrast, CO-PtO_5_ loses CO_2_ to form PtO_4_ with an energy barrier of 0.81 eV. It should be noted that the formed PtO_4_ here has a similar structure as square planar Pt_1_ on the CeO_2_ step site (Vii), so the step-site situation is not considered individually. PtO_4_ can either adsorb O_2_ to close cycle 2 or adsorb CO to form CO-PtO_4_, which will further transform to PtO_3_ after CO_2_ removal. PtO_3_ then adsorbs CO strongly, but adsorbed CO-PtO_3_ is unlikely to transform to PtO_2_ due to its endothermic nature. Instead, it adsorbs O_2_ with an adsorption energy of −2.09 eV to complete cycle 3, which prevents the sintering of Pt_1_. The overall energy barriers of relatively favorable cycle 2 in adsorbed Pt_1_ in Pt/CeO_2_ and square planar Pt_1_ in Pt_AT_CeO_2_ are 0.69 and 0.81 eV, respectively. Such a small difference should not induce the huge activity difference in Fig. 3, which also implies parts of Pt_1_ in Pt/CeO_2_ sinter under CO oxidation condition. Supplementary Fig. S33 shows the simulated CO vibrational frequencies on both adsorbed and square planar Pt_1_. The calculated vibrational frequencies of adsorbed CO on PtO_3_ for both Pt_1_ structures are consistent with the CO-DRIFTS results, indicating that the observed CO-Pt_1_ band in CO-DRIFTS can be attributed to the adsorbed CO on PtO_3_. What sets Pt/CeO_2_ apart is that PtO_3_ further transforms into PtO and then sinters at higher temperatures. However, PtO_3_ is relatively stable in Pt_AT_CeO_2_.

It has been reported that Ce vacancy (V_Ce_) is more difficult to generate compared to the V_O_^37^. However, we can speculate that the exsolution of square planar Pt_1_ on the CeO_2_ terrace in Pt_AT_CeO_2_ is a strategy to generate V_Ce_. The possible reason for the formation of square planar Pt_1_ only at 800 °C is the lattice expansion of CeO_2_ at high temperatures, as seen in in-situ XRD (Supplementary Fig. S34) and the favorable migration of cerium cations to PGMs surface at higher temperatures in O_2_^38–40^. These could facilitate the surface CeO_2_ reconstruction around the Pt atom or migration of cerium cations onto the Pt surface to form a local square planar Pt_1_ structure (Supplementary Fig. S35). We then construct two models for Pt/CeO_2_-CO and Pt_AT_CeO_2_-CO with a five-atom Pt NP on CeO_2_ (111) surface with and without V_Ce_ (Supplementary Fig. S36) to simulate their redispersion process. This process includes the oxidation of the five-atom Pt NP to the PtO_x_ cluster and the redispersion of the top Pt atom (Supplementary Fig. S37). The results indicate that the redispersion of the top Pt atom into a surrounding V_Ce_ is energetically more favorable than the intact CeO_2_ surface (formation energy: −2.02 eV vs 0.26 eV). Therefore, from a thermodynamic point of view, V_Ce_ generated after the exsolution of square planar Pt_1_ could, in return, trap Pt atoms more readily during a reoxidation treatment to form a square planar structure again. In contrast, Pt clusters are formed without surface V_Ce_ after reducing the adsorbed Pt_1_ on CeO_2_, and then it redisperses into adsorbed form after a reoxidation treatment. This explains why two SACs have the memory to return to their respective native structure. The different dynamic evolution under CO oxidation condition and their structural memory behaviors under reductive-oxiditive treatment cycle is due to their various initial Pt_1_ location on CeO_2_ driven by different calcination temperatures. Therefore, designing SACs with tunable location is important to maximize their catalytic performance in the future.

In summary, Pt/CeO_2_ and Pt_AT_CeO_2_ were fabricated via two different annealing temperatures of 500 and 800 °C. Pt atoms are both atomically dispersed in nascent Pt/CeO_2_ and Pt_AT_CeO_2_, evidenced by the combined characterization results. These two catalysts display dramatically different catalytic activity toward CO oxidation and different apparent activation energies and reaction orders in CO and O_2_. These differences could be explained by the different initial Pt_1_ local configurations, where Pt_1_ in Pt/CeO_2_ and Pt_AT_CeO_2_ are dominated by adsorbed Pt_1_ and square planar Pt_1_, respectively. Under reaction condition, adsorbed Pt_1_ in Pt/CeO_2_ sinters into few-atom Pt clusters; however, square planar Pt_1_ in Pt_AT_CeO_2_ is strongly anchored to the support with a decrease in the Pt-O coordination number. After the treatment in CO at 275 °C, both types of Pt_1_ transform to Pt NPs, which inevitably redisperse at the elevated temperature in O_2_ or even under O_2_-rich reaction condition. What is more interesting is that the initial thermal treatment creates memory on the support where the Pt atoms return under CO oxidation or oxidative conditions, potentially providing a catalyst self-healing after severe catalyst sintering.

## Methods

### Synthesis of Pt/CeO_2_ and Pt_AT_CeO_2_

CeO_2_ powder was synthesized by the precipitation method with ammonia, followed by washing with DI water, drying, and calcination at 500 °C in air for 4 h. Tetraammineplatinum(II) nitrate was then impregnated on CeO_2_ powder by the incipient wetness impregnation, with the calculated Pt weight loadings of 1%. After impregnation, the samples were dried at 100 °C for 12 h, followed by calcination at 500 °C and 800 °C in air for 10 h to yield Pt/CeO_2_ and Pt_AT_CeO_2_ catalysts, respectively. Pt/CeO_2_-CO and Pt_AT_CeO_2_-CO were obtained after treating the fresh samples in CO/Ar (20 mL/min) at 275 °C for 20 min. Pt/CeO_2_-CO-O_2_ and Pt_AT_CeO_2_-CO-O_2_ were achieved after further treating the reduced samples in air at 500 °C for 10 h. The low-loading catalysts were synthesized by the same method.

### Activity measurements

CO oxidation experiments of fresh Pt/CeO_2_ and Pt_AT_CeO_2_ were carried out in a fixed-bed flow reactor. Then, 20 mg of catalyst sieved between 40 and 80 mesh was diluted with 380 mg washed SiC powder and then loaded together into the reactor tube. The reaction temperature was ramped up from 20 to 500 °C with a heating rate of 3 °C /min in the mixture of 1 mL/min CO, 4 mL/min O_2_, and 95 mL/min Ar, with a weight hourly space velocity (WHSV) of 300 L/g*h. The reactor was cooled down to 20 °C in the above reaction mixture for the next light-off test. The product concentration was measured by a gas chromatograph Agilent 3000 Micro GC. The activity measurements of reduced and reoxidized catalysts were performed under the same reaction condition after the in situ pretreatment in the same fixed-bed flow reactor. CO oxidation kinetic measurements were carried out under different reaction conditions by controlling the CO conversion lower than 8%. The partial pressures of CO and O_2_ were adjusted by changing their flow rates. To study the effect of CO partial pressure on reaction rate, the partial pressure of O_2_ was kept at 4 kPa, and the partial pressure of CO changed between 0.6 and 3 kPa. To study the effect of O_2_ partial pressure on reaction rate, the partial pressure of CO was kept at 1 kPa, and the partial pressure of O_2_ changed between 1 and 10 kPa. The reported reaction rates were normalized by the total numbers of Pt, assuming that all Pt are accessible.

### Characterization

Powder X-ray diffraction (XRD) patterns were collected using a Rigaku Miniflex 600 equipped with Cu Kα radiation, with an operating voltage of 40 kV and a current of 15 mA. All samples are collected from 15 to 65° with a speed of 0.5°/min. In situ XRD was carried out in an XRD cell on a PANalytical Empyrean X-ray diffractometer equipped with Cu Kα radiation, with an operating voltage of 45 kV and a current of 40 mA. Quasi in situ X-ray photoelectron spectroscopy measurements were carried out with a Physical Electronics Quantera SXM Scanning X-ray Microprobe with a focused monochromatic Al Kα X-ray (1486.7 eV) source and multi-channel detector. Prior to the test, the samples were pretreated in a preparation chamber under different temperatures and gases, i.e., 180 °C in CO and O_2_ or 275 °C in CO. After the pretreatment, the samples were directly transferred into the XPS detection chamber for the test without exposure to other gases. All spectra, including Pt, Ce, and O in binding energies, were charge corrected by shifting the Ce^4+^ 3d_5/2_ line to 916.7 eV^41^. Diffuse-reflectance infrared Fourier transform spectroscopy with CO as the probe molecule (CO-DRIFTS) was carried out on a Thermo Scientific IS-50R FTIR with the MCT/A detector. Prior to analysis, approximately 40 mg of the sample was pretreated at 200 °C for 30 min with O_2_/He flow in a DRIFTS reaction chamber. A spectral resolution of 4 cm^−1^ was used to collect spectra, and each spectrum in the work is an average of 32 scans. Ex situ XAS measurements were performed at X-ray Science Division bending-magnet beamline at sector 20 of the Advanced Photon Source operating at Argonne National Laboratory. In brief, the samples after calcination were pressed and covered into thin sheets in air before the test. In situ XAS measurements were carried out at the Stanford Synchrotron Radiation Light Source (SSRL) at beamline 9-3 in fluorescence mode. The catalysts were characterized by in situ XAS at the Pt *L*_*3*_-edge (11564 eV) using an in-house built cell with a 4-mm ID glassy carbon tube. The catalyst and standard samples were scanned simultaneously in transmission and fluorescence detection modes using ion chambers and a 100-element solid-state Ge monolith detector (Canberra). XANES and EXAFS data processing and analysis were performed using Athena and Artemis programs of the Demeter data analysis package^42,43^. The detailed measurement and analysis methods can be seen in our previous study^21^. The theoretical EXAFS signals for the Pt–O path of Pt_1_ adsorbed on CeO_2_ were generated using the FEFF6 code from a Pt doped on the CeO_2_ model. The theoretical EXAFS signals were fitted to the data in R-space using Artemis by varying the coordination numbers of the single scattering paths, the effective scattering lengths, the bond length disorder, and the correction to the threshold energy, ΔE_0_ (common for all paths since they are all from the same FEFF calculation). S_0_^2^ (the passive electron reduction factor) was obtained by first analyzing the spectrum for the Pt oxide, and the best-fit value (0.90) was fixed in the fit. The k-range used for fitting was 3–14 Å^−1^ while the R-range was 1.2–2 Å for the model that only includes the Pt-O scattering shell. High-angle annular dark-field scanning transmission electron microscopy (HAAD-STEM) images were collected on a Nion UltraSTEM microscope operated at 100 keV. Inductively coupled plasma-atomic emission spectroscopy (ICP-AES) was performed using an Optima 2100 DV spectrometer (PerkinElmer Corporation). N_2_ adsorption-desorption isotherms were analyzed at 77 K on the Micromeritics gas adsorption apparatus (Quadrasorb-EVO, Quantachrome Corporation, America). Prior to analysis, all samples were pretreated at 200 °C for 4 h in a vacuum condition. The specific surface area was calculated using the Brunauer–Emmett–Teller (BET) equation. Temperature-programmed desorption of CO was performed on Micromeritics Autochem 2920 with a TCD detector and coupled mass spectroscopy (MS) detector. Prior to analysis, the sample was pretreated at 500 °C in He for 30 min, followed by cooling down to room temperature in He. The treated sample was then exposed to 10% CO/Ar before ramping in He. Temperature-programmed surface reaction (TPSR) was carried out with the same instrument. The visible Raman spectra (532 nm) were collected on a Horiba LabRAM HR Raman/FTIR microscope equipped with a Synapse Charge Coupled Device (CCD) camera and an in situ sample cell (Linkam CCR 1000). All Raman spectra were conducted at room temperature, including the one after CO pretreatment at 275 °C. No obvious changes upon extended laser exposure were observed in the sample.

### DFT calculations

The periodic density function theory (DFT) calculations were carried out with the CP2K package^44^. The generalized-gradient approximation (GGA) with Perdew−Burke−Ernzerhof (PBE) functional was used to evaluate the exchange and correlation^45^. The wave functions were expanded in a molecularly optimized double-Gaussian basis set, with an auxiliary plane wave basis set with a cutoff energy of 500 Rydberg. The scalar relativistic norm-conserving pseudo-potentials were employed to model the core electrons^46^ with 18, 12, and 6 valence electrons for Pt, Ce, and O, respectively. The only Γ-point in the reciprocal space mesh was used for integrating the Brillouin zone. The DFT + U method^47^, based on the Mullikan 4f state population analysis, was used to describe the Ce 4f electrons. A U value was set at ~4.1 eV in line with the previous literature^48^, which ensures that the redox property is reproduced correctly^49^. Grimme’s third-generation DFT-D3 approach was used to describe dispersion corrections^50^. The CeO_2_(111) surfaces were used to model the CeO_2_ substrate, constructed with cell dimensions of 15.344 × 13.288 × 27.529 Å with 15-Å vacuum space to minimize the interaction between slabs. Geometry optimization was performed based on the BFGS method. The convergence criterion used for geometry optimizations was a maximum force of 0.01 eV Å^−1^. Spin polarization was considered in all calculations.

## Supplementary information

Supplementary Information

Peer Review File

## Data Availability

The data generated in this study are provided in the Supplementary Information. More detailed data that support the findings of this study are available from the corresponding author upon reasonable request.
