# Supplementary material for: Memory-dictated dynamics of single-atom Pt on CeO2 for CO oxidation
Source: Nat Commun. 2023 May 9;14:2664. doi: 10.1038/s41467-023-37776-3 (PMC10169862; doi:10.1038/s41467-023-37776-3)
Supplement: Supplementary file 1 — Supplementary Information [file 41467_2023_37776_MOESM1_ESM.pdf]

Supplementary Information

Memory-dictated Dynamics of Single-atom Pt on CeO_2 for CO Oxidation

Zihao Zhang,[1,2,†] Jinshu Tian,[1,†] Yubing Lu,[1] Shize Yang,[3] Dong Jiang,[2] Weixin Huang,[2] Yixiao Li,[2] Jiyun Hong,[4] Adam S. Hoffman,[4] Simon R. Bare,[4] Mark H. Engelhard,[1] Abhaya K. Datye,[5] Yong Wang*,[1,2]

[1] Institute for Integrated Catalysis, Pacific Northwest National Laboratory, Richland, WA 99354, USA

[2] The Gene and Linda Voiland School of Chemical Engineering and Bioengineering, Washington State University, Pullman, WA 99164, USA

[3] Eyring Materials Center, Arizona State University, Tempe, AZ 85257, USA

[4] Stanford Synchrotron Radiation Light Source, SLAC National Accelerator Laboratory, Menlo Park, CA 94025, USA

[5] Department of Chemical and Biological Engineering and Center for Micro-Engineered Materials, University of New Mexico, Albuquerque, NM 87131, USA

[†] These authors contributed equally

*Corresponding author. Email: yong.wang@pnnl.gov (Y. Wang)

Content

Supplementary Tables S1-S3

Supplementary Figures S1-S37

Supplementary Tables

Table S1 The actual Pt loadings and surface atomic percentage (Pt, Ce, and O) determined by ICP-AES and XPS, respectively

Samples	Pt (wt%)	Surface at. Pt%	Surface at. Ce%	Surface at. O%
CeO_2	~	~	27.5	72.5
Pt/CeO_2	0.75	0.8	26.6	72.6
$Pt_{AT}CeO_2$	0.81	0.9	26.0	73.1
Pt/CeO_2-180 °C[a]	~	0.7	14.7	84.6
$Pt_{AT}CeO_2$-180 °C[a]	~	1.2	19.8	79.1
CeO_2-CO	~	~	25.5	74.5
Pt/CeO_2-CO	~	0.4	25.2	74.5
$Pt_{AT}CeO_2$-CO	~	0.6	25.5	73.9

[a]Pt/CeO_2-180 °C and $Pt_{AT}CeO_2$-180 °C represent the catalysts after treatment at 180 °C under CO oxidation condition for 20 mins before test.

Table S2 Best-fit EXAFS models characterizing results at the Pt L_3-edge for the Pt/CeO_2 and $Pt_{AT}CeO_2$ in ambient air at 25 °C, and CO oxidation condition at 25 °C and 180 °C. R: radial distance. CN: coordination number. E_0: correction to the threshold energy, σ^2: Debye-Waller-like term

Samples	Path	R (Å)	CN	σ^2 ×10^3 (Å^2)	E_0 (eV)	R factor
Pt/CeO_2-ambient air	Pt-O	2.00 ± 0.01	5.0 ± 0.4	2 ± 1	4.5 ± 1.1	0.003
$Pt_{AT}CeO_2$-ambient air	Pt-O	2.00 ± 0.01	4.9 ± 0.5	2 ± 1	3.7 ± 1.5	0.005
Pt/CeO_2-CO/O_2 25°C	Pt-O	2.00 ± 0.03	3.1 ± 0.9	3 ± 3	6.3 ± 3.7	0.008
$Pt_{AT}CeO_2$-CO/O_2 25°C	Pt-O	2.00 ± 0.02	4.8 ± 1.2	4 ± 3	5.3 ± 3.2	0.006
Pt/CeO_2-CO/O_2 180°C	Pt-O	2.00 ± 0.02	2.8 ± 0.7	4 ± 3	6.2 ± 3.2	0.006
$Pt_{AT}CeO_2$-CO/O_2 180°C	Pt-O	2.00 ± 0.01	3.2 ± 0.6	3 ± 2	5.6 ± 2.2	0.003

Table S3 BET surface area, pore volume, pore size, and average crystallite size of different samples determined by N_2 adsorption-desorption isotherms and XRD

Samples	BET surface area (m^2/g)	Pore volume (cm^3/g)	Pore size (nm)	Crystallite size (nm)
CeO_2	53.3	0.34	20.1	11.5
800CeO_2	13.4	0.11	34.1	31.5
0.1Pt/800CeO_2	14.2	0.12	33.9	32.3
0.1Pt_{AT}800CeO_2	13.5	0.13	38.0	32.0
0.1Pt/CeO_2	53.6	0.26	19.5	~
0.1$Pt_{AT}CeO_2$	13.8	0.16	46.8	~
Pt/CeO_2	51.1	0.26	20.3	12.0
Pt/CeO_2-CO	~	~	~	12.2
Pt/CeO_2-CO-O_2	~	~	~	12.3
$Pt_{AT}CeO_2$	27.2	0.16	23.9	21.7
$Pt_{AT}CeO_2$-CO	~	~	~	21.8
$Pt_{AT}CeO_2$-CO-O_2	~	~	~	20.5

Supplementary Figures

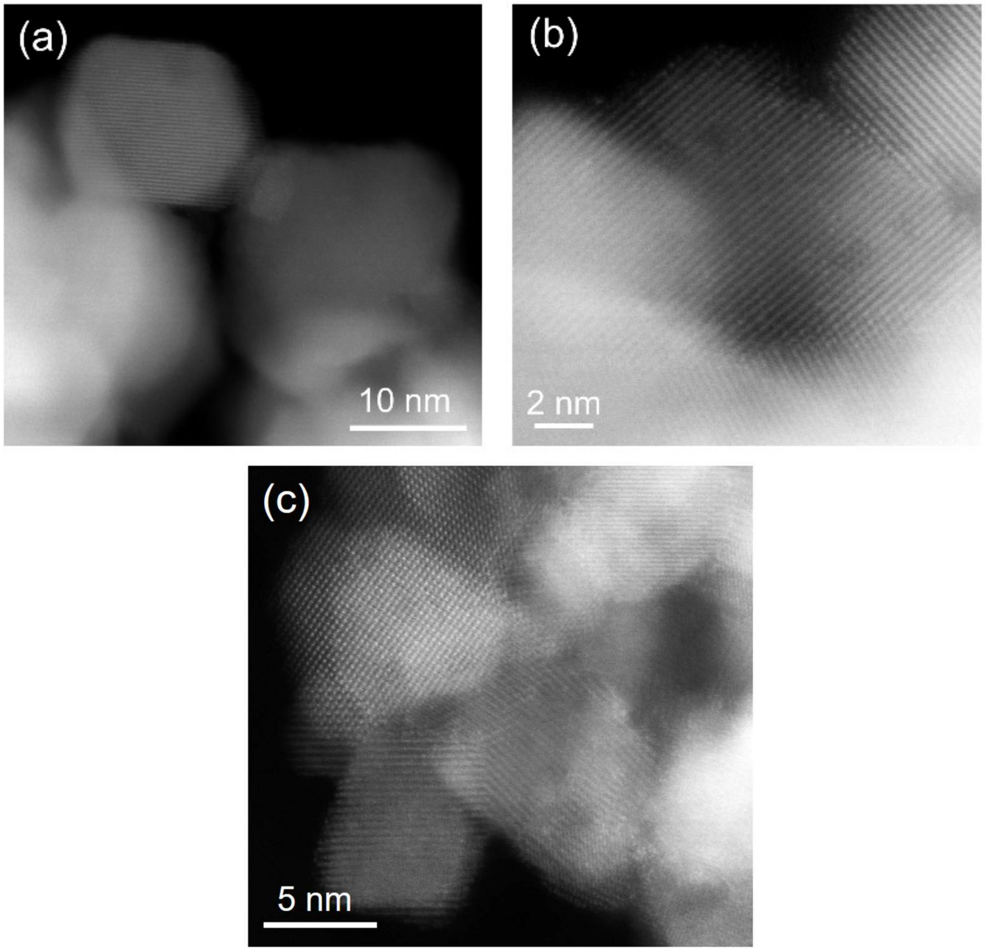

Fig. S1 HAADF-STEM images of (a,b) $Pt_{AT}CeO_2$ and (c) Pt/CeO_2

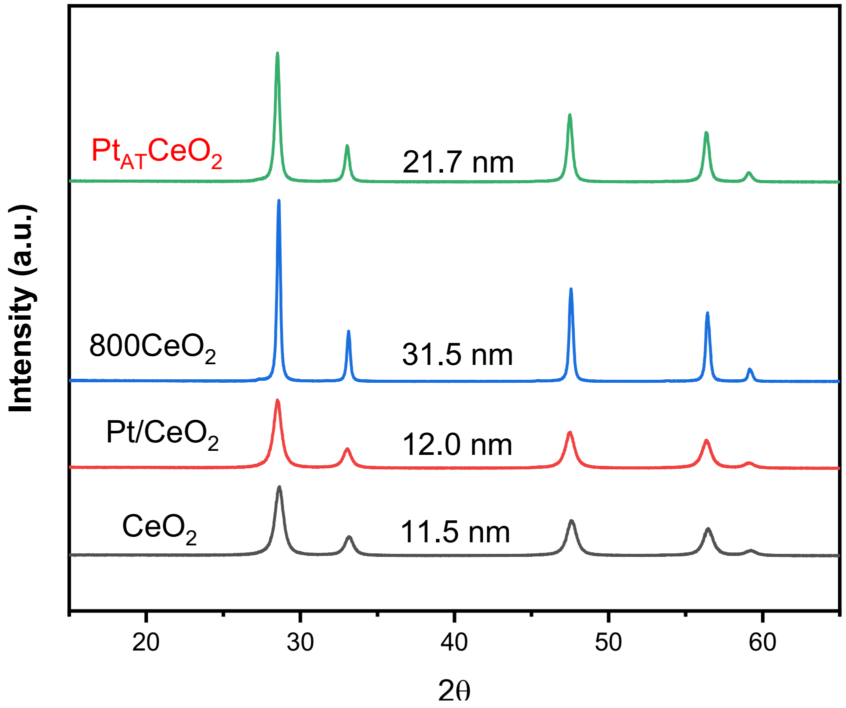

Fig. S2 Powder XRD patterns of CeO_2 (calcined at 500 °C), Pt/CeO_2, and $Pt_{AT}CeO_2$. $800CeO_2$ is calcined directly at 800 °C for 10 h. The average CeO_2 crystallite sizes determined by Scherrer equation from CeO_2 (111) peak were also included

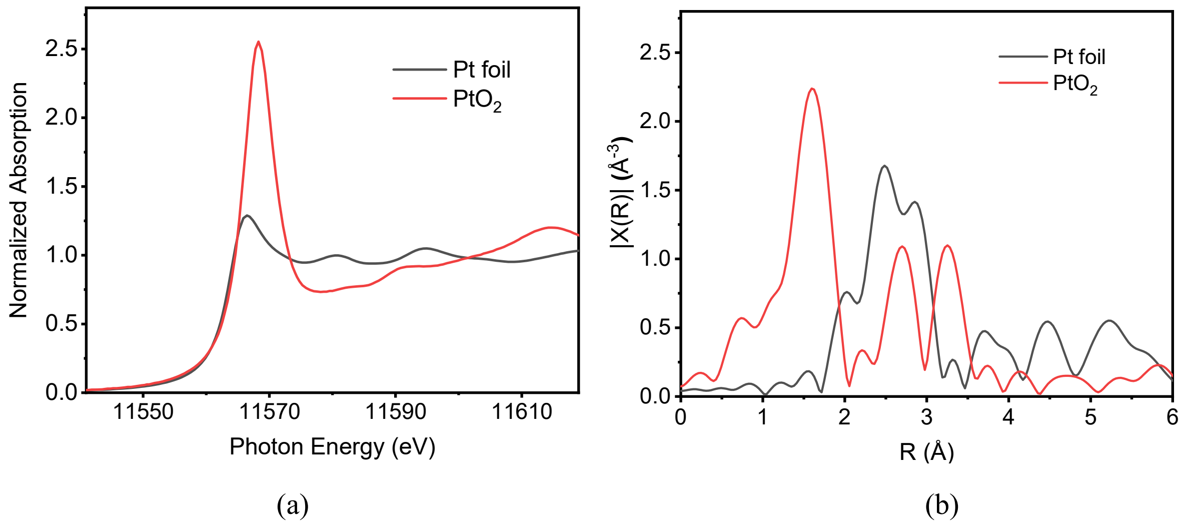

Fig. S3 Pt L_3-edge XANES and the corresponding EXAFS spectra of references Pt foil and PtO_2

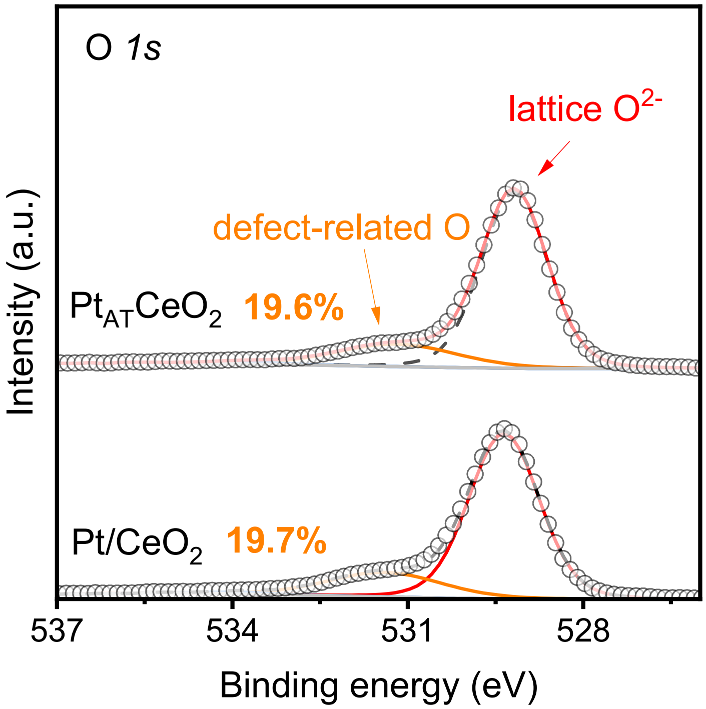

Fig. S4 O *1s* XPS spectra of Pt/CeO_2 and $Pt_{AT}CeO_2$

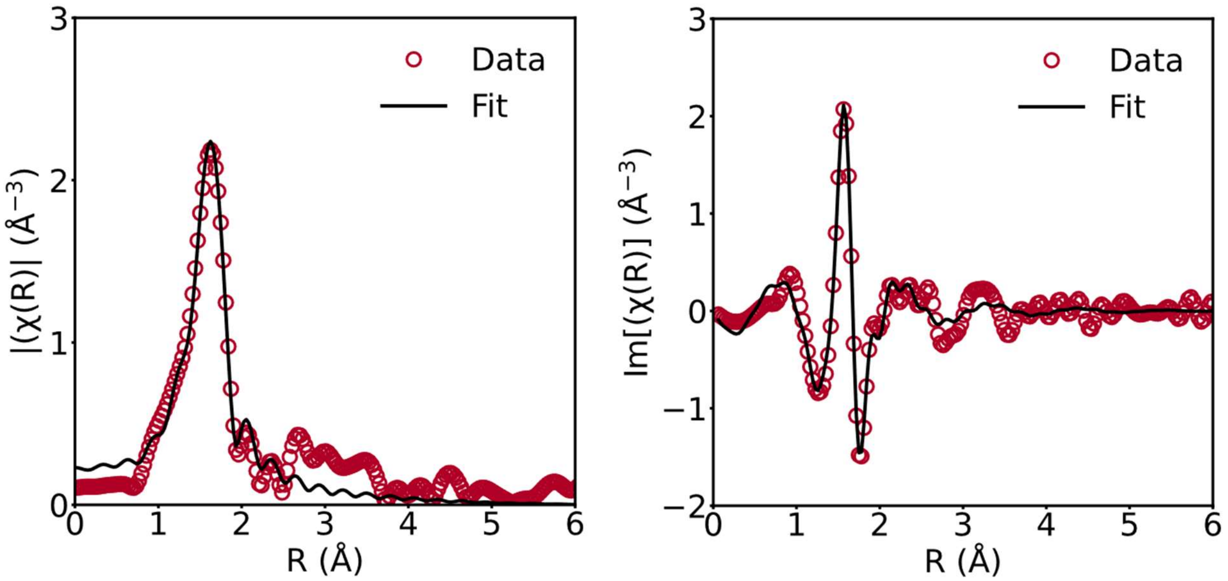

Fig. S5 Pt L_3-edge X-ray absorption spectroscopy of fresh Pt/CeO_2. Magnitude (left) and imaginary component (right) of the Fourier-transform of the χ(k) data of fresh Pt/CeO_2 collected under air. Data (circle) and model fit (solid line). R= 1.2-2 Å for the fit window. Δk = 3-14 $Å^{-1}$ for the Fourier transform

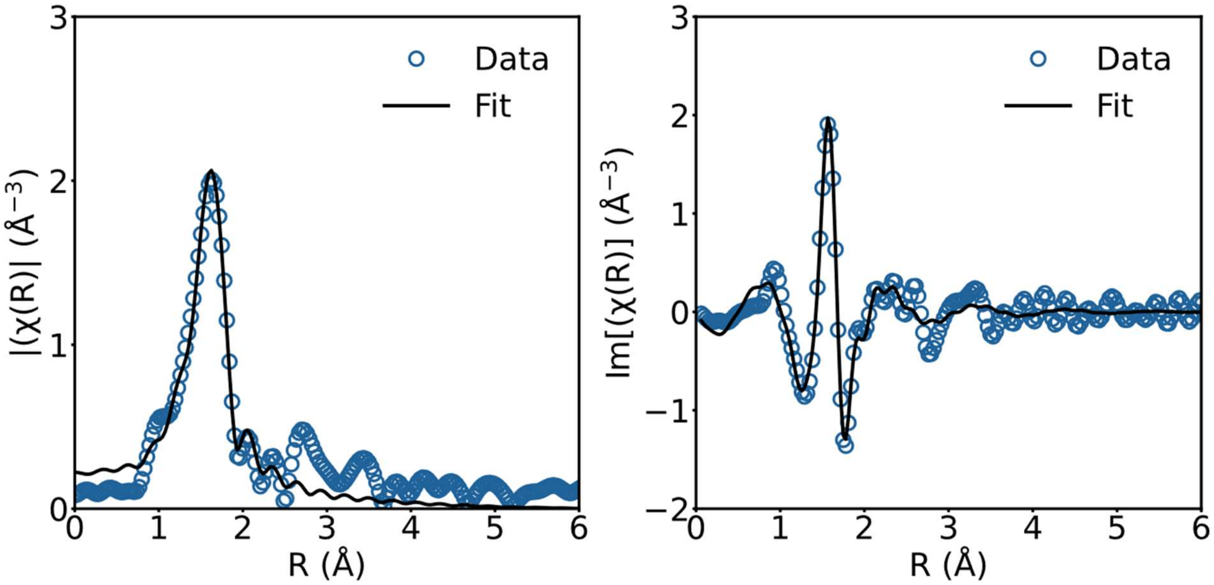

Fig. S6 Pt L_3-edge X-ray absorption spectroscopy of fresh $Pt_{AT}CeO_2$. Magnitude (left) and imaginary component (right) of the Fourier-transform of the χ(k) data of fresh $Pt_{AT}CeO_2$ collected under air. Data (circle) and model fit (solid line). R= 1.2-2 Å for the fit window. Δk = 3-14 Å^{-1} for the Fourier transform

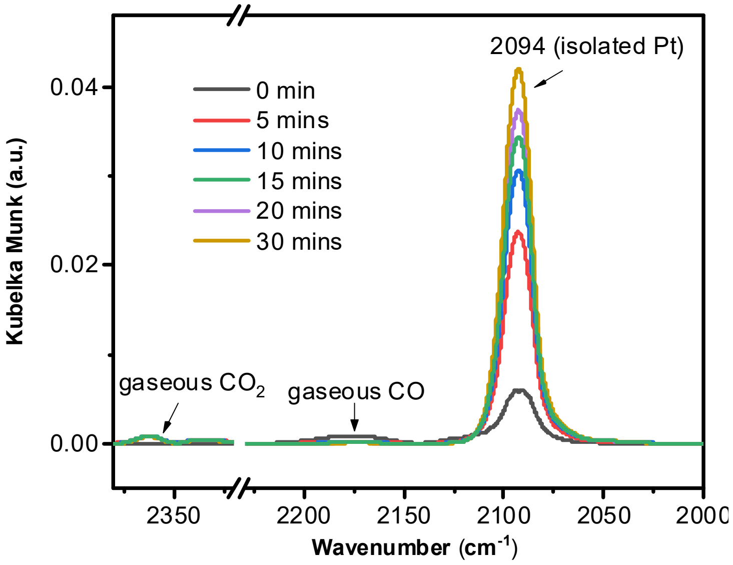

(a)

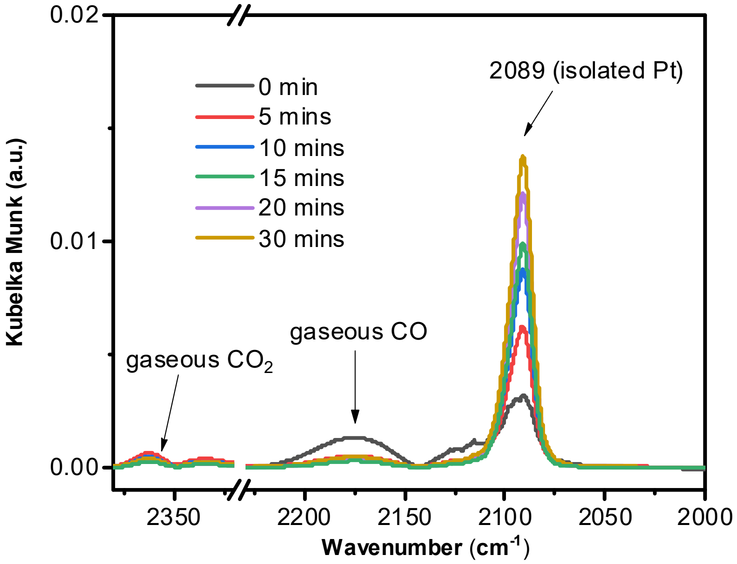

(b)

Fig. S7 *In situ* CO diffuse reflectance infrared Fourier transform spectra (CO-DRIFTs) for (a) Pt/CeO_2 and (b) $Pt_{AT}CeO_2$ at 100 °C in the flowing 1% CO and 8% O_2 mixture (balanced in He) from 0 ~ 30 mins. Operation condition: After increasing temperature to 100 °C in O_2/He, 1% CO was introduced and the spectra were recorded for the next 30 mins

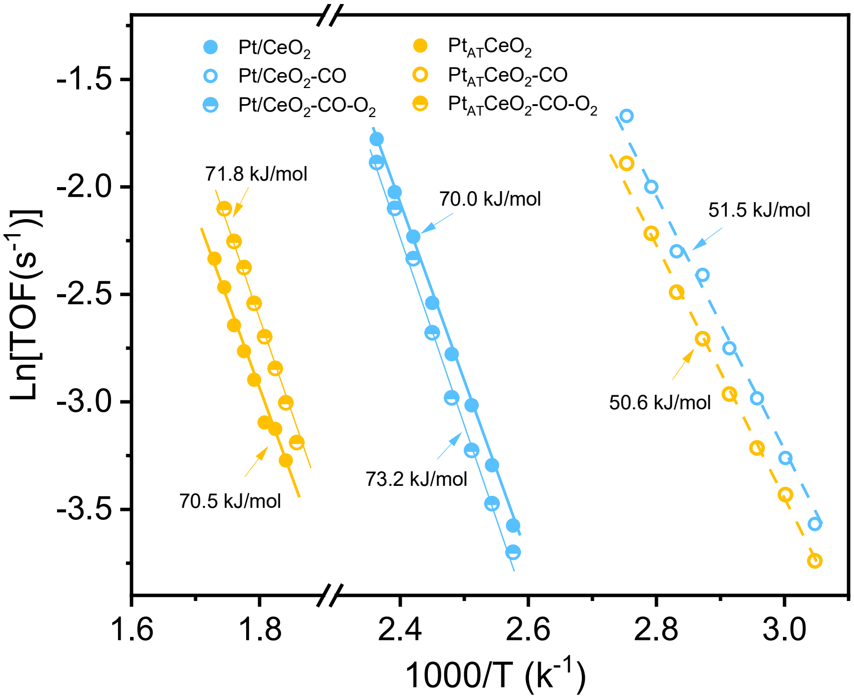

Fig. S8 Arrhenius plots of fresh, CO-reduced, and re-oxidized Pt/CeO_2 and $Pt_{AT}CeO_2$. 1% CO and 4% O_2 balanced in Ar with WHSV of 300 L/g*h, 20 mg catalyst diluted with SiC to 400 mg

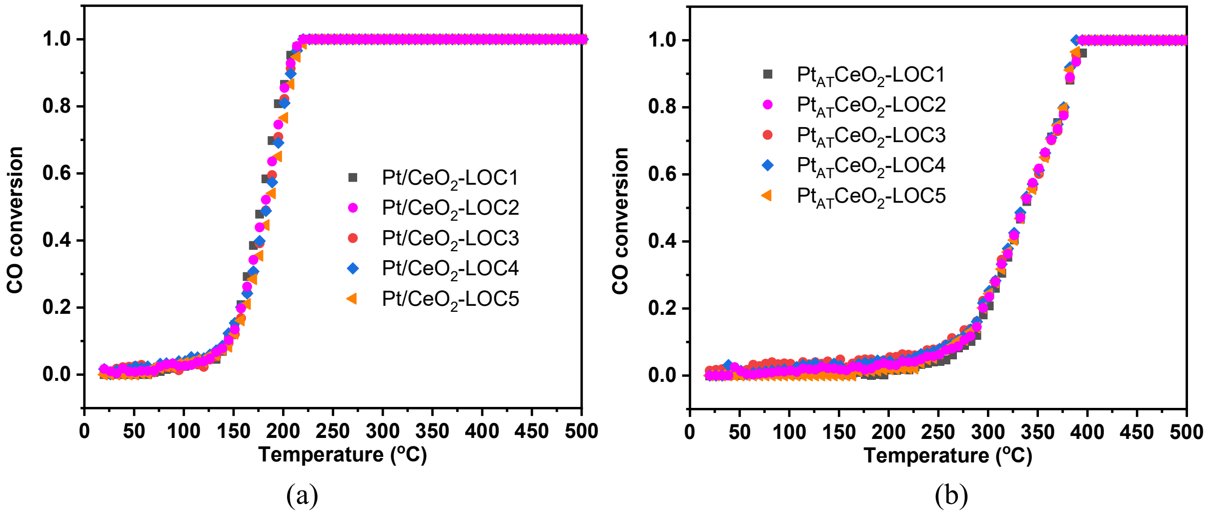

Fig. S9 Five repeated light-off curves (LOCs) of fresh (a) Pt/CeO_2 and (b) $Pt_{AT}CeO_2$. Reaction conditions: catalyst loading = 20 mg, diluted with SiC to 400 mg, 1% CO and 4% O_2 balanced in Ar, total flow rate = 100 mL/min, ramping from 20 to 500 °C with the ramping rate of 3 °C/min for each cycle; For instance, Pt/CeO_2-LOC2 represents the second light-off curve of Pt/CeO_2; $Pt_{AT}CeO_2$-LOC4 represents the fourth light-off curve of $Pt_{AT}CeO_2$

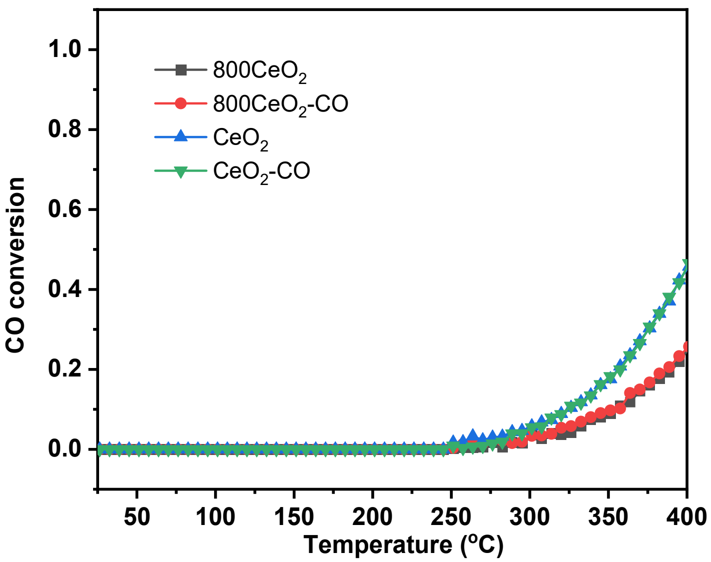

Fig. S10 CO oxidation performance (light-off curve) of CeO_2 (precalcined at 500 °C) and 800CeO_2 (precalcined at 800 °C) before and after CO reduction. Reaction conditions: catalyst loading = 20 mg, diliuted with SiC to 400 mg, 1% CO and 4% O_2 balanced in Ar, total flow rate = 100 mL/min, ramping from 20 to 400 °C with the ramping rate of 3 °C/min for each cycle

800CeO_2 shows slightly lower CO oxidation reactivity than CeO_2 (calcined at 500 °C), that is ascribed to its lower surface area (53.3 m^2/g for CeO_2 and 13.4 m^2/g for 800CeO_2, **Table S3**). In addition, the CO oxidation reactivity of pure CeO_2 and 800CeO_2 does not change even after CO pretreatment.

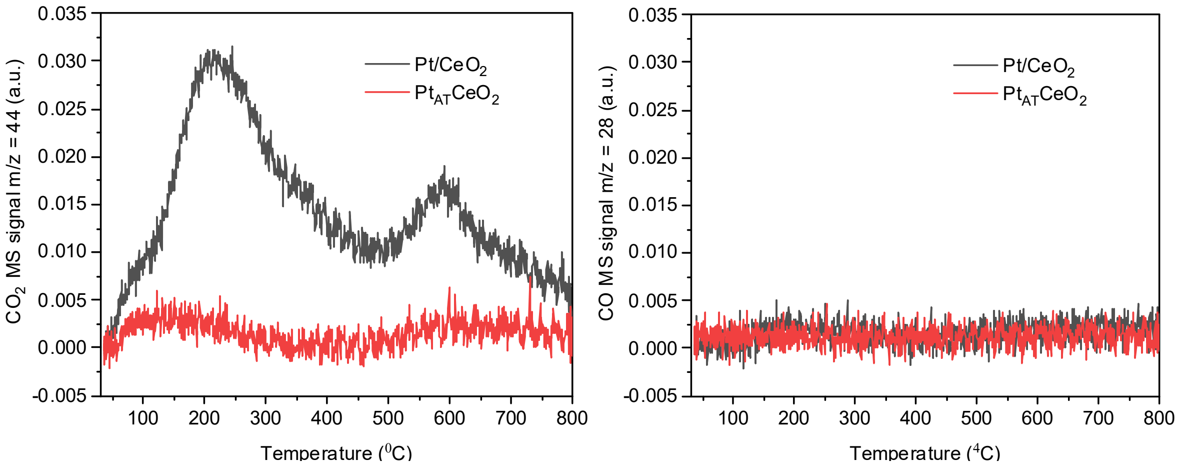

(a) (b)

Fig. S11 (a) CO_2 MS signal (m/z = 44) and (b) CO MS signal (m/z = 28) during temperature programmed desorption of CO from 35 to 800 °C on Pt/CeO_2 and $Pt_{AT}CeO_2$. During the CO desorption process, only CO_2 was monitored with very little CO signal being detected, suggesting that adsorbed CO will react with lattice O of CeO_2 to form CO_2 before desorption

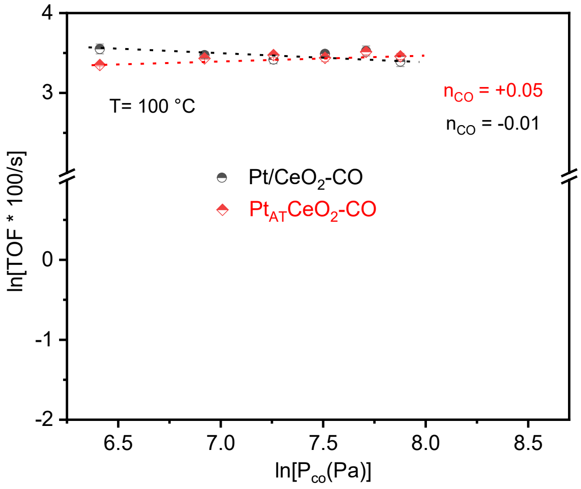

(a)

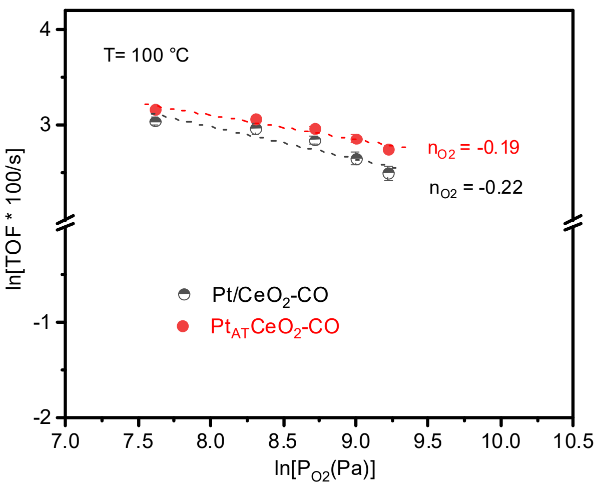

(b)

Fig. S12 (a) CO and (b) O_2 partial pressure on the reaction rate (TOF) of Pt/CeO_2-CO and $Pt_{AT}CeO_2$-CO, respectively. The reaction temperature is chosen as 100 °C. Two reduced catalysts show similar CO oxidation activity, and similar reaction orders in both CO and O_2 due to the formation of Pt aggregates after reduction

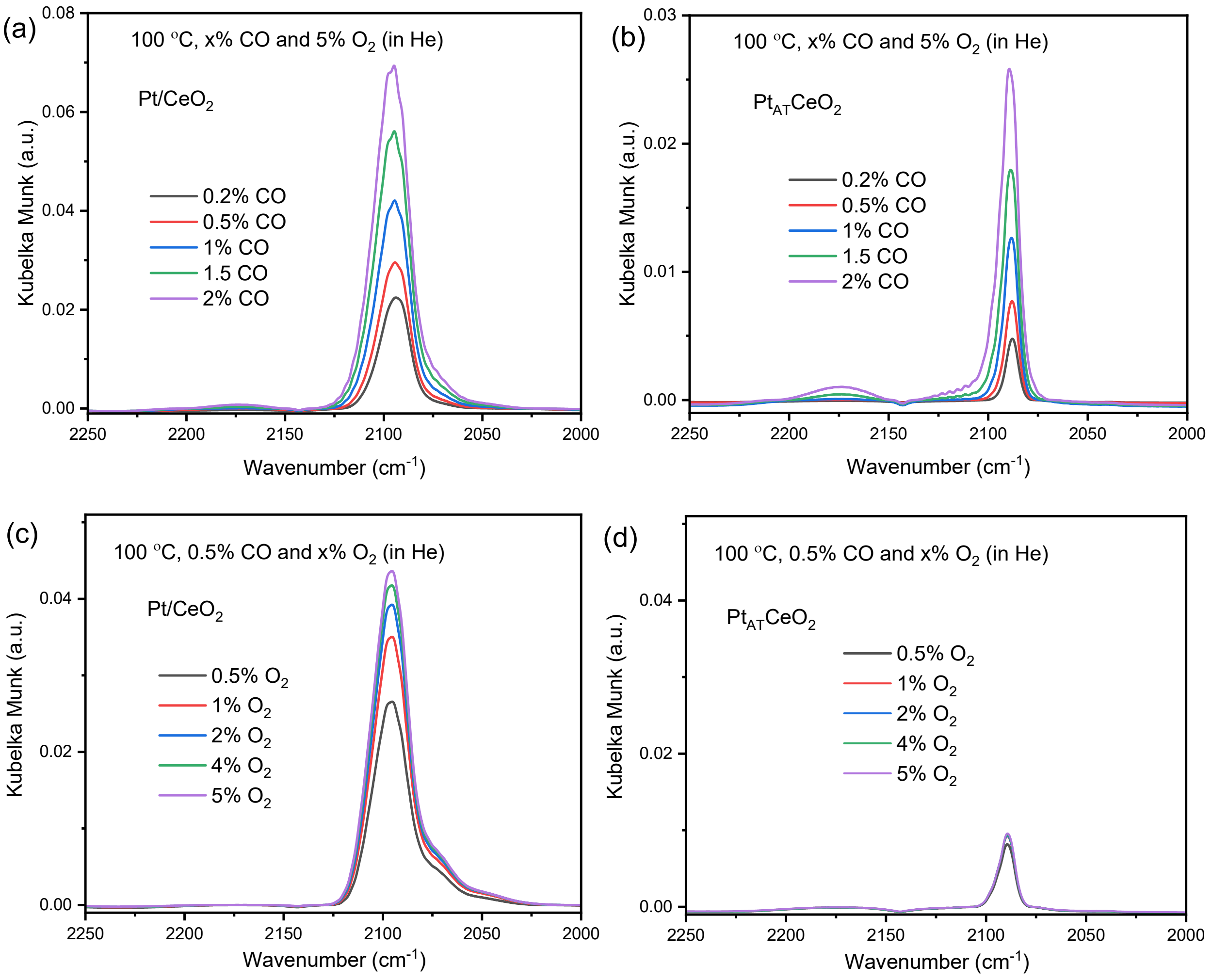

Fig. S13 CO-DRIFTS of (a,c) Pt/CeO_2 and (b,d) $Pt_{AT}CeO_2$, the effect of (a,b) CO and (c,d) O_2 partial pressures on the surface coverage of Pt_1 species at 100 ºC. For (a,b) CO-dependent experiments, CO partial pressure increases from 0.2% to 2% in sequence with 5% O_2. For (c,d) O_2-dependent experiments, O_2 partial pressure increases from 0.5% to 5% in sequence with 0.5% CO

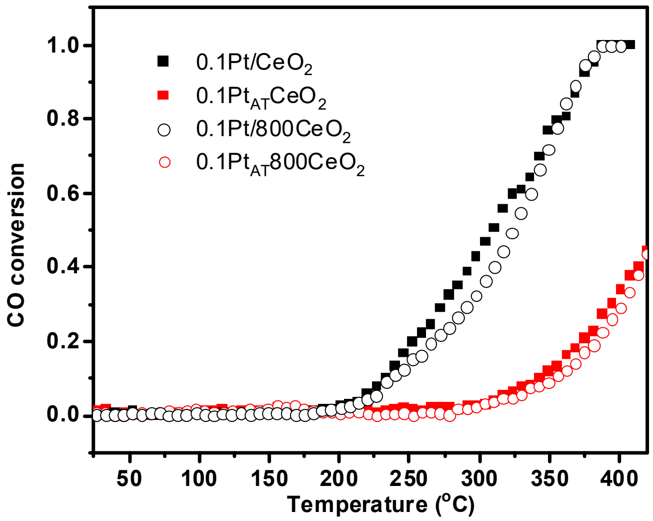

Fig. S14 CO oxidation performance (light-off curve) of 0.1Pt/CeO_2, 0.1$Pt_{AT}CeO_2$, 0.1Pt/800CeO_2, and 0.1Pt_{AT}800CeO_2. Reaction conditions: catalyst loading = 20 mg, diluted with SiC to 400 mg, 1% CO and 4% O_2 balanced in Ar, total flow rate = 100 mL/min, ramping from 20 to 400 °C with the ramping rate of 3 °C/min for each cycle

The average CeO_2 crystallite sizes of Pt/CeO_2 and $Pt_{AT}CeO_2$ are 12.0 and 21.7 nm (**Table S3**), as determined by the Scherrer equation of XRD patterns (**Fig. S2**). The surface areas of Pt/CeO_2 and $Pt_{AT}CeO_2$ determined by N_2 adsorption-desorption isotherms (**Fig. S16**, **Table S3**) are 51.1 and 27.2 m^2/g, respectively. Pt/CeO_2 has larger surface area and smaller CeO_2 size than $Pt_{AT}CeO_2$. To exclude the effect of surface area and CeO_2 grain size on reactivity, CeO_2 support was pre-calcined at 800 °C for 10 h to sacrifice the CeO_2 surface through particle agglomeration. A small amount of Pt (0.1 wt%) was then impregnated on the above-synthesized 800CeO_2 support to maintain the atomically dispersed nature, followed by calcination at 500 and 800 °C to obtain 0.1Pt/800CeO_2 and 0.1Pt_{AT}800CeO_2, respectively. Because of the pre-calcination of the support at 800 °C, 0.1Pt/800CeO_2 and 0.1Pt_{AT}800CeO_2 exhibited similar porosity properties (**Table S3**, **Fig. S17**) and CeO_2 particle size (**Fig. S18**) to the 800CeO_2 support. The obtained 0.1Pt/800CeO_2 still showed much higher CO oxidation activity than 0.1Pt_{AT}800CeO_2 (**Fig. S14**), although they have very similar surface area and CeO_2 grain size. To rule out the influence of few Pt clusters present in Pt/CeO_2 on activity, 0.1Pt/CeO_2 and 0.1$Pt_{AT}CeO_2$ with a low Pt loading (~0.1 wt%) were prepared, which also showed the similar activity trend (**Fig. S14**). At such a low Pt loading, it is unlikely for oxidized Pt clusters to exist in fresh 0.1Pt/CeO_2 after calcination at 500 °C in air.

It should be noted that CO conversion is almost 100% over Pt/CeO_2 at 200 °C (**Fig. 3a**), but only around 2% over 0.1Pt/CeO_2. If the initial Pt_1 structure in Pt/CeO_2 was the real active site, we should expect around 10% conversion over 0.1Pt/CeO_2 at 200 °C. The much lower reactivity in 0.1Pt/CeO_2 might further prove that the *in situ* formed Pt clusters are the real active sites in Pt/CeO_2, since the lower surface Pt_1 density in 0.1Pt/CeO_2 is less likely to sinter under the same condition.

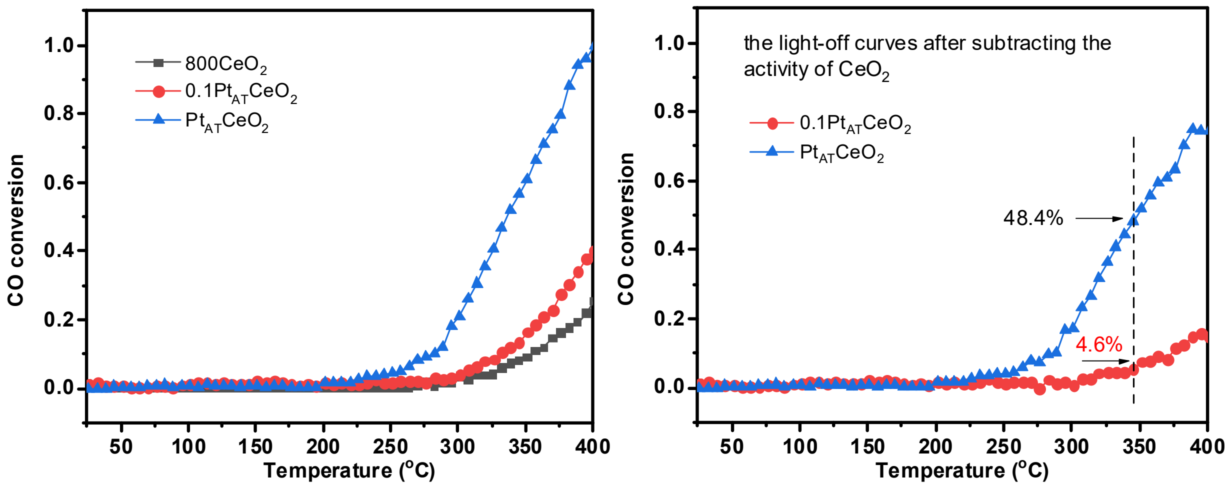

(a) (b)

Fig. S15 (a) CO oxidation performance of $800CeO_2$ (calcined at 800 °C), $0.1Pt_{AT}CeO_2$, and $Pt_{AT}CeO_2$; (b) CO oxidation performance of $0.1Pt_{AT}CeO_2$, and $Pt_{AT}CeO_2$ after subtracting the activity of CeO_2.

Because of the similar activity of $0.1Pt_{AT}CeO_2$ and pure CeO_2, the effect of support on the reactivity should be discussed. We have combined the light-off curves for $800CeO_2$, $Pt_{AT}CeO_2$ and $0.1Pt_{AT}CeO_2$ (**Fig. S15a**). Apparently, the catalytic activities of $Pt_{AT}CeO_2$ and $0.1Pt_{AT}CeO_2$ are still higher than that of pure CeO_2. Even with only 0.1 wt% Pt on CeO_2 surface, the difference in activity is evident, suggesting that the square-planar Pt_1 in $Pt_{AT}CeO_2$ is indeed more active than CeO_2. However, CeO_2 does contribute to some activity above 300 °C. To exclude the influence of CeO_2, the light-off curves of $Pt_{AT}CeO_2$ and $0.1Pt_{AT}CeO_2$ were obtained by simply subtracting the activity of CeO_2, as seen in **Fig. S15b**. The CO conversion over $Pt_{AT}CeO_2$ is roughly 10 times higher than that of $0.1Pt_{AT}CeO_2$, for example, 48.4% and 4.6% at 347 °C, respectively (**Fig. S15b**). This further suggests the Pt_1 in $Pt_{AT}CeO_2$ does not transform into Pt cluster, otherwise we will see a more than tenfold activity difference.

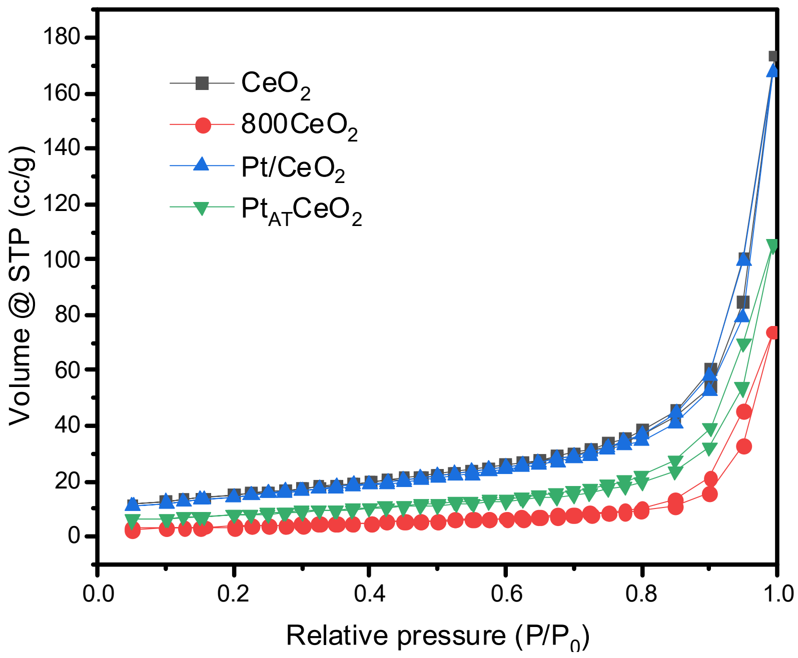

Fig. S16 N_2 adsorption-desorption isotherms at 77 K of CeO_2, $800CeO_2$, Pt/CeO_2, and $Pt_{AT}CeO_2$

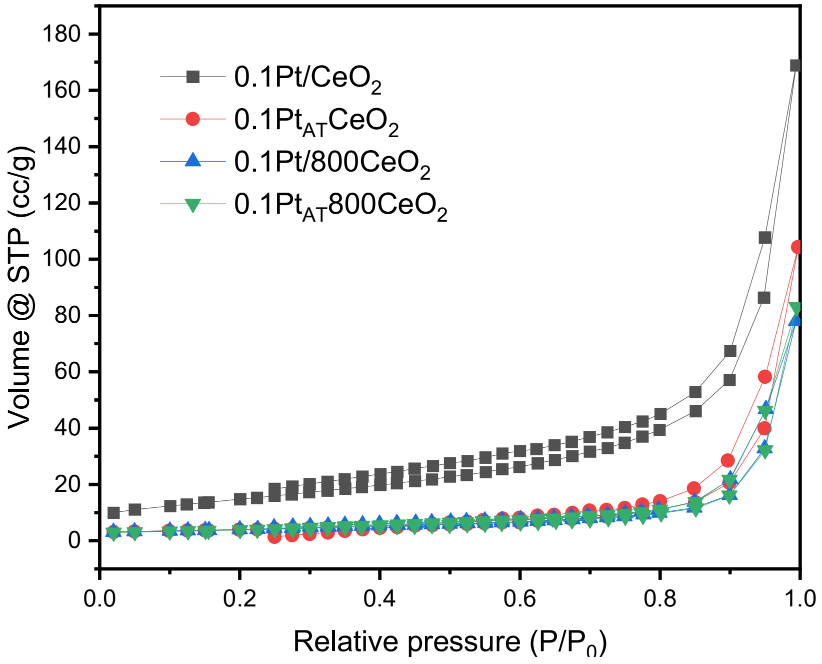

Fig. S17 N_2 adsorption-desorption isotherms at 77 K of 0.1Pt/CeO_2, 0.1$Pt_{AT}CeO_2$, 0.1Pt/800CeO_2, and 0.1Pt_{AT}800CeO_2

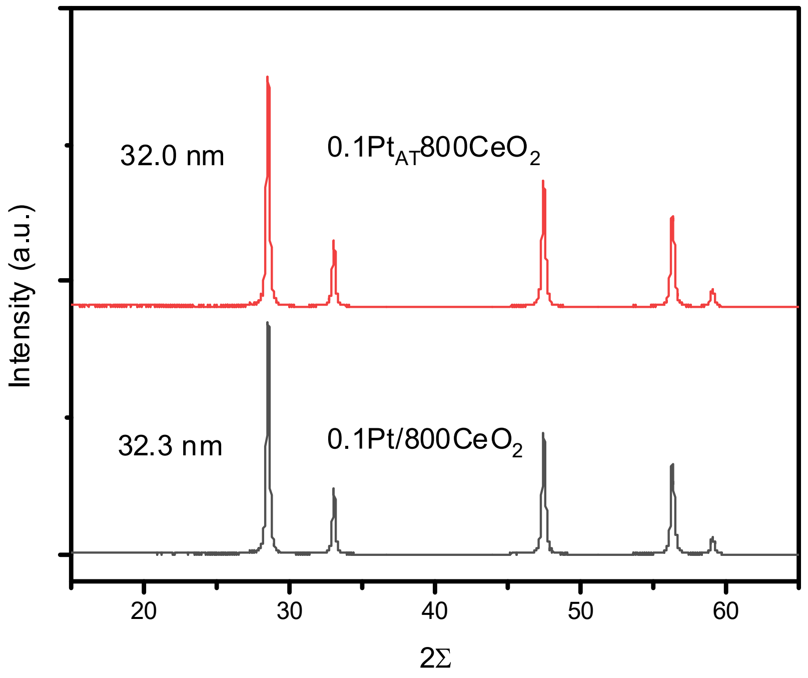

Fig. S18 Powder XRD patterns of 0.1Pt/800CeO_2, and 0.1Pt$_{AT}$800CeO_2. These two samples show very similar CeO_2 crystallite size

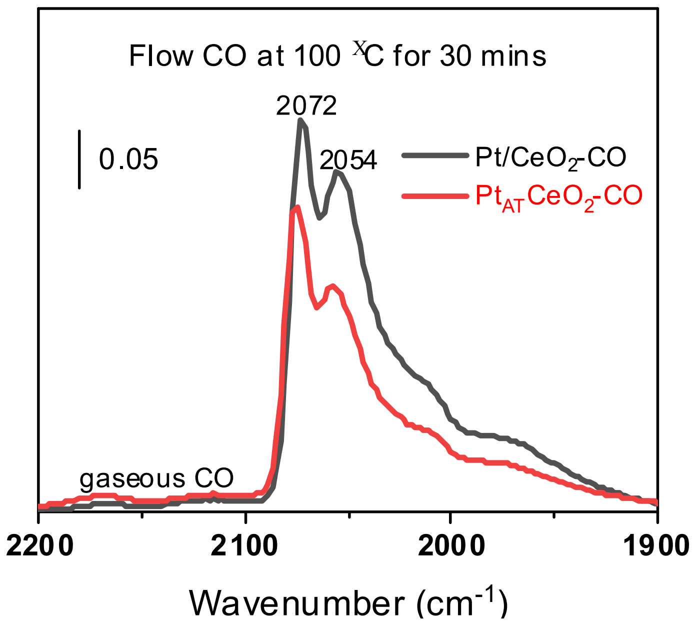

Fig. S19 CO-DRIFTs of Pt/CeO_2-CO and $Pt_{AT}CeO_2$-CO. Operation condition: after pretreatment of fresh sample in CO at 275 °C for 20 mins, the sample was cooled down to 100 °C in CO before test.

By combining other characterization techniques, the infrared features around 2072 and 2054 cm^{-1} can be attributed to the vibrations of CO adsorbed on the Pt NPs. For example, the formation of Pt NPs can be clearly confirmed by the TEM images (Fig. 5a,5d), XPS spectra (Fig. S23), and Raman spectra (Fig. S24).

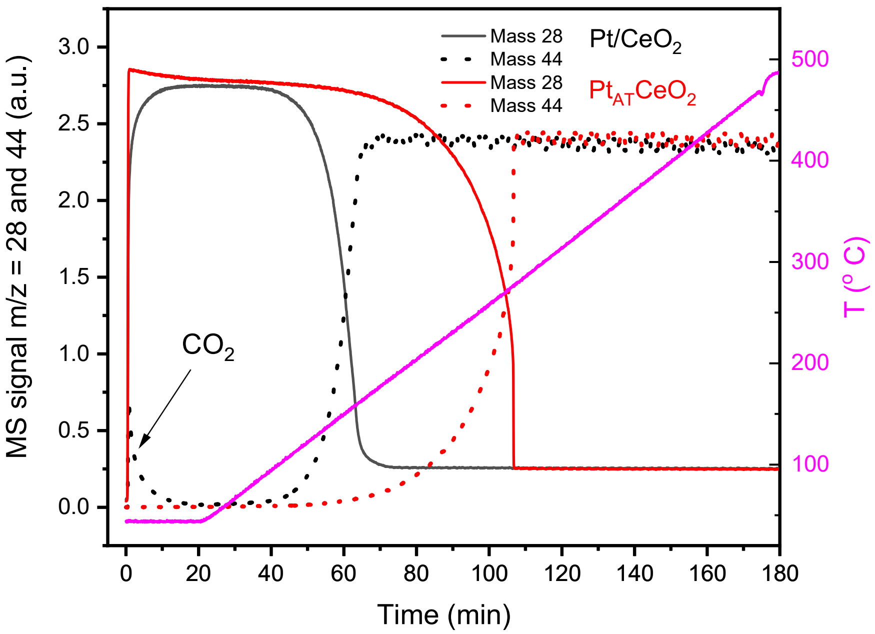

Fig. S20 MS signal m/z = 28 (CO) and 44 (CO_2) intensity during temperature programmed surface reaction (TPSR). Before 0 min, the sample was pretreated in O_2 at 500 °C for 30 mins, then cool down to room temperature (RT) in O_2. At 0 min, CO is introduced into O_2 flow with CO/O_2 ratio of 1:4 at RT. After 20 mins, the temperature increased to 500 °C with the heating rate of 3 °C/min to simulate the CO oxidation process. The reactivity result is consistent with light-off curve performance in Figure 3a

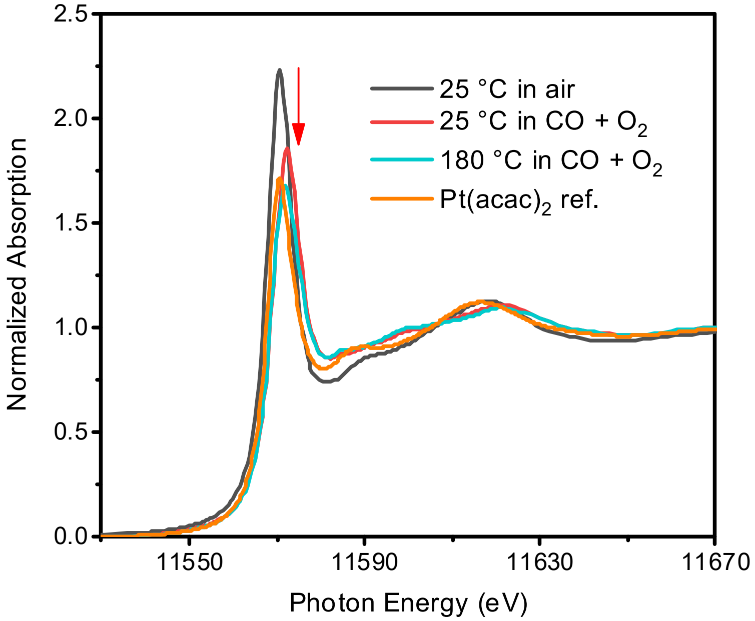

Fig. S21 Pt L_3-edge XANES of Pt/CeO_2 at 25 °C in ambient air, at 25 °C and 180 °C in CO oxidation condition, as well as the Pt(acac)$_2$ (Pt^{2+}) reference

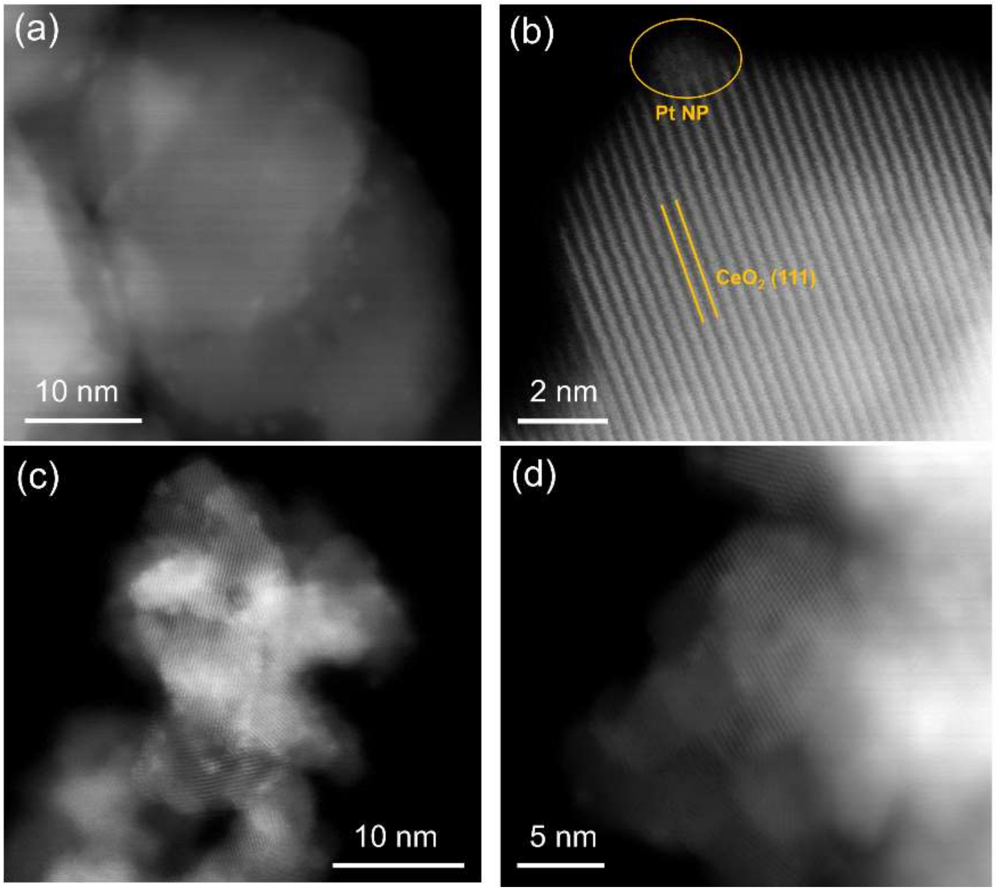

Fig. S22 HAADF-STEM images of (a, b) $Pt_{AT}CeO_2$-CO and (c, d) Pt/CeO_2-CO

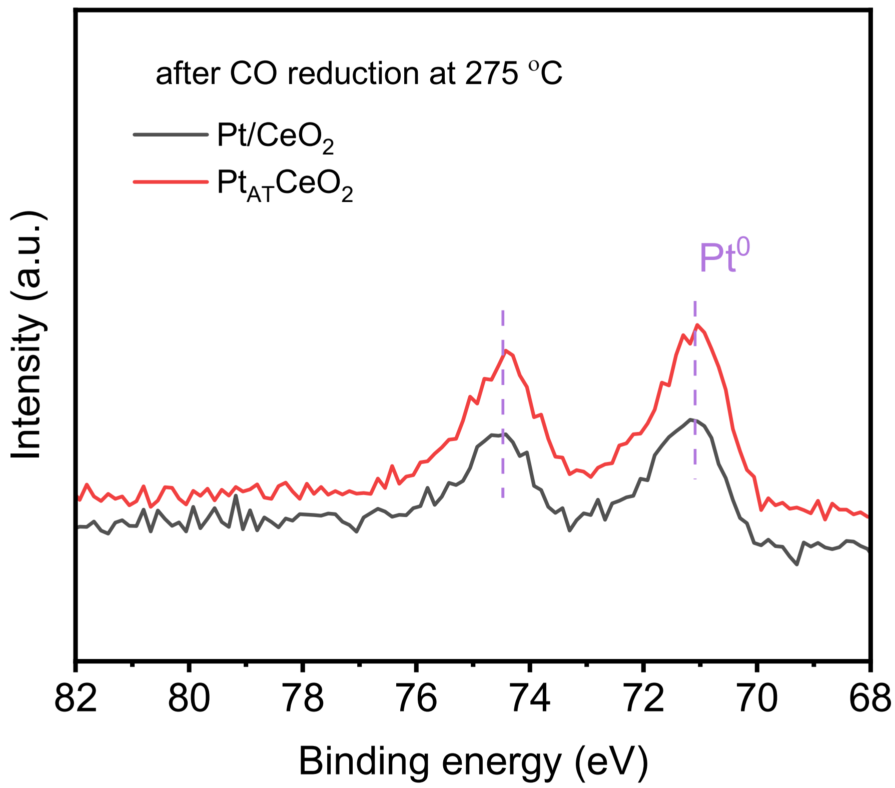

Fig. S23 Pt *4f* XPS spectra for Pt/CeO_2 and $Pt_{AT}CeO_2$ after treatment in CO at 275 °C for 20 mins

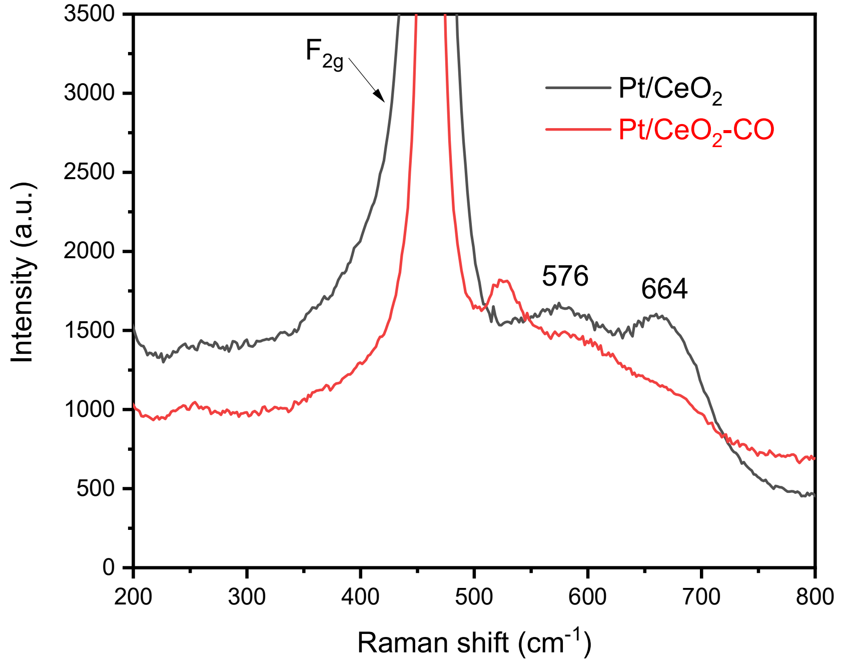

(a)

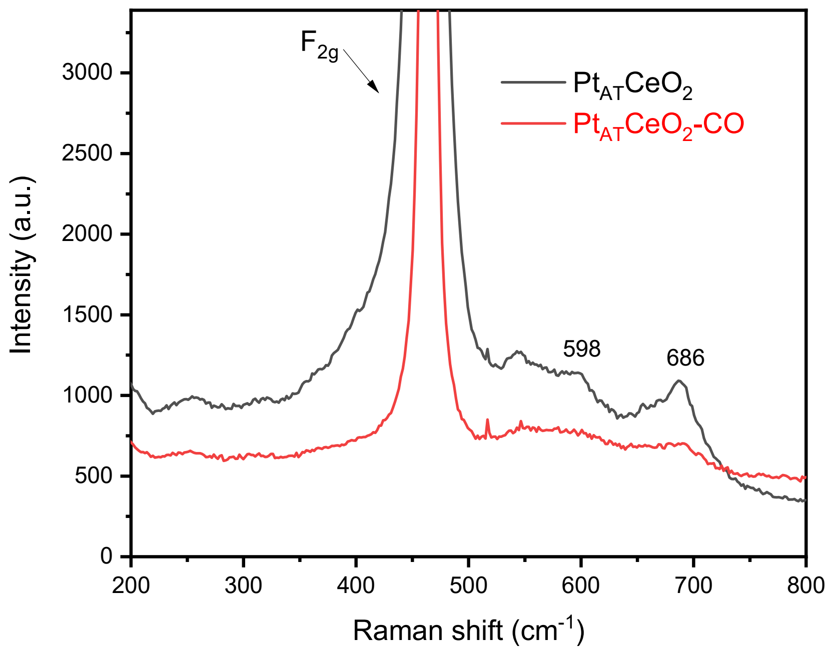

(b)

Fig. S24 Raman spectra of fresh and CO-reduced (a) Pt/CeO_2 and (b) $Pt_{AT}CeO_2$. Raman spectra of Pt/CeO_2-CO and $Pt_{AT}CeO_2$-CO were collected after *in situ* reduction at 275 °C with CO in Raman cell. The distinct bands at 576 and 667 cm^{-1} for Pt/CeO_2 and 598 and 686 cm^{-1} for $Pt_{AT}CeO_2$ are usually considered as the single-atom Pt features ascribed from the Pt-O or Pt-O-Ce vibrations. After the CO reduction, the Raman intensity of those peaks significantly decrease, which also suggest the formation of Pt NPs in Pt/CeO_2-CO and $Pt_{AT}CeO_2$-CO.

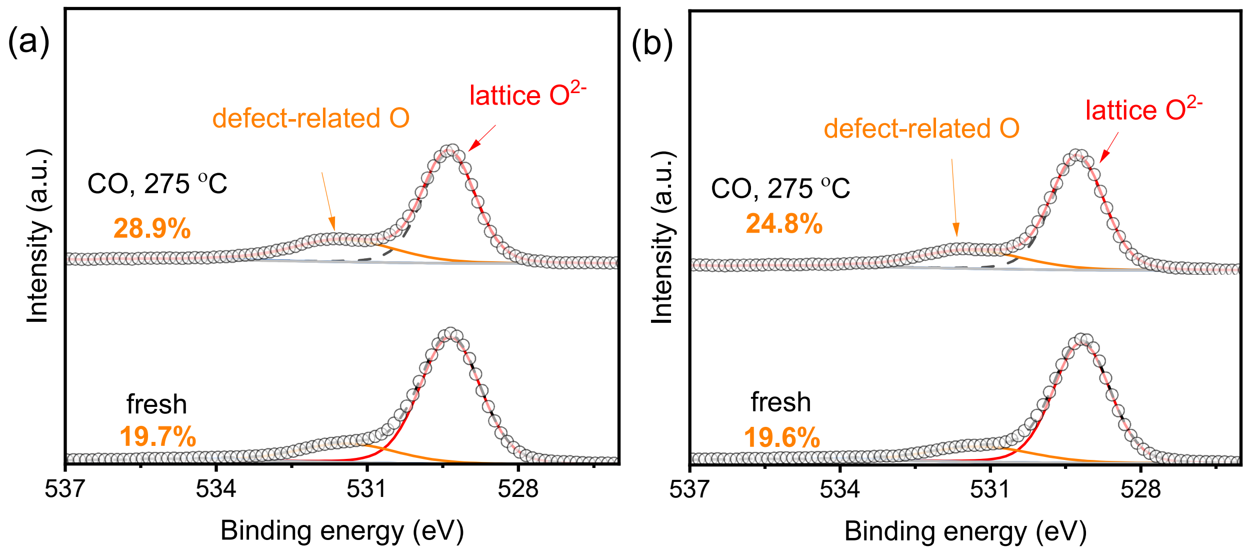

Fig. S25 O*1s* XPS spectra of fresh and reduced (a) Pt/CeO_2 and (b) $Pt_{AT}CeO_2$ samples. The peak at ~529.3 eV is attributed to the lattice O, and the peaks at ~531.4 eV and 534.3 eV are ascribed to two types of defect-related O.

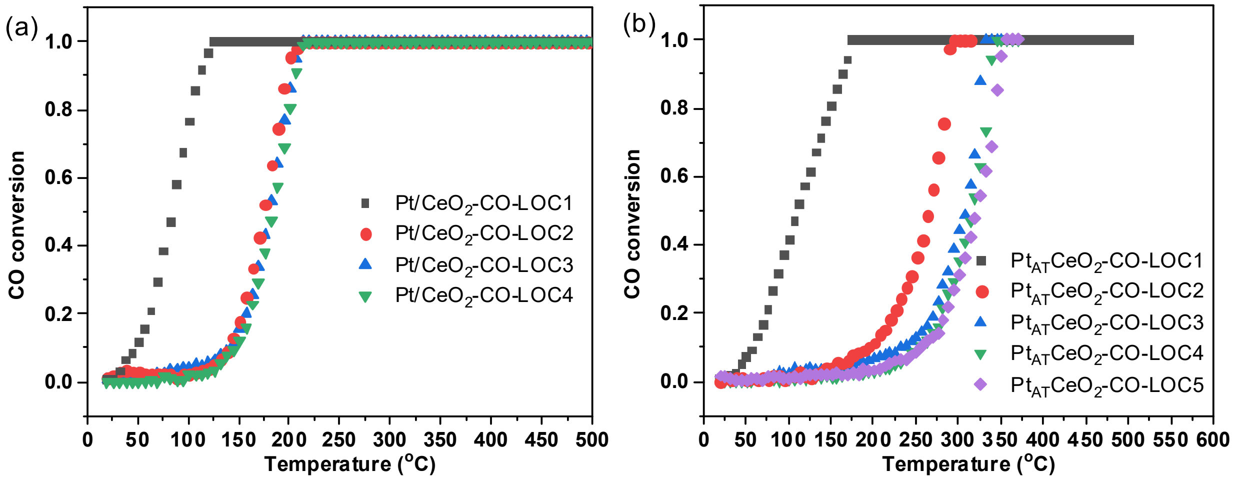

Fig. S26 Multiple light-off curves of (a) Pt/CeO_2-CO and (b) $Pt_{AT}CeO_2$-CO samples. Each light-off curve was performed from 25 to 500 °C. For instance, Pt/CeO_2-CO-LOC2 represents the second light-off curve of Pt/CeO_2-CO; $Pt_{AT}CeO_2$-CO-LOC4 represents the fourth light-off curve of $Pt_{AT}CeO_2$-CO

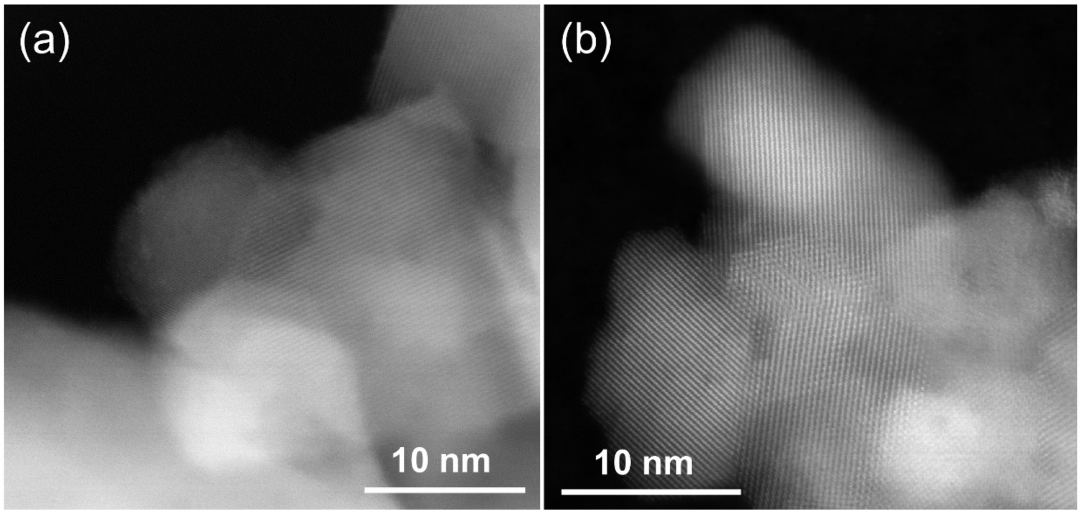

Fig. S27 HAADF-STEM images of (a) $Pt_{AT}CeO_2$-CO-O_2 and (b) Pt/CeO_2-CO-O_2

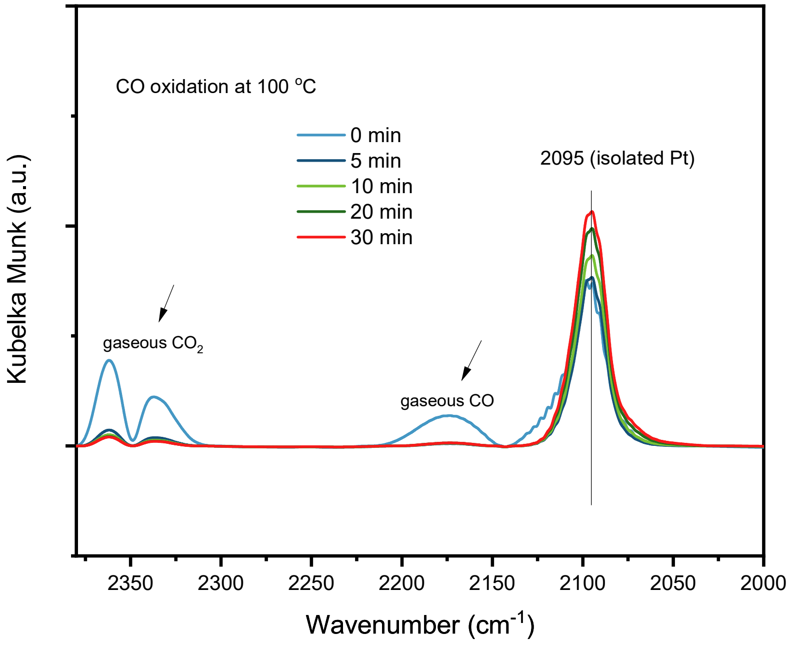

Fig. S28 *In situ* CO-DRIFTs of Pt/CeO_2-CO-O_2 at 100 °C under CO oxidation condition up to 30 mins.

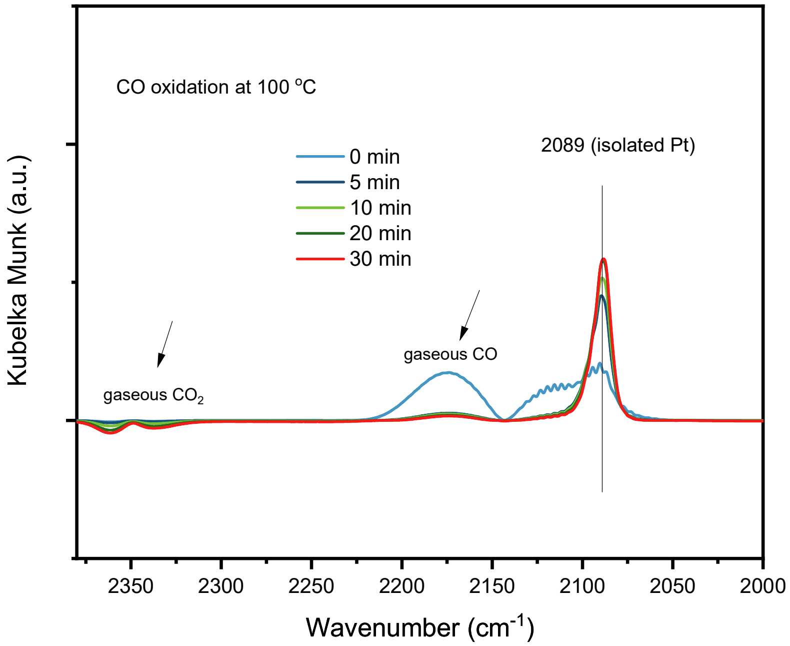

Fig. S29 *In situ* CO-DRIFTs of $Pt_{AT}CeO_2$-CO-O_2 at 100 °C under CO oxidation condition up to 30 mins

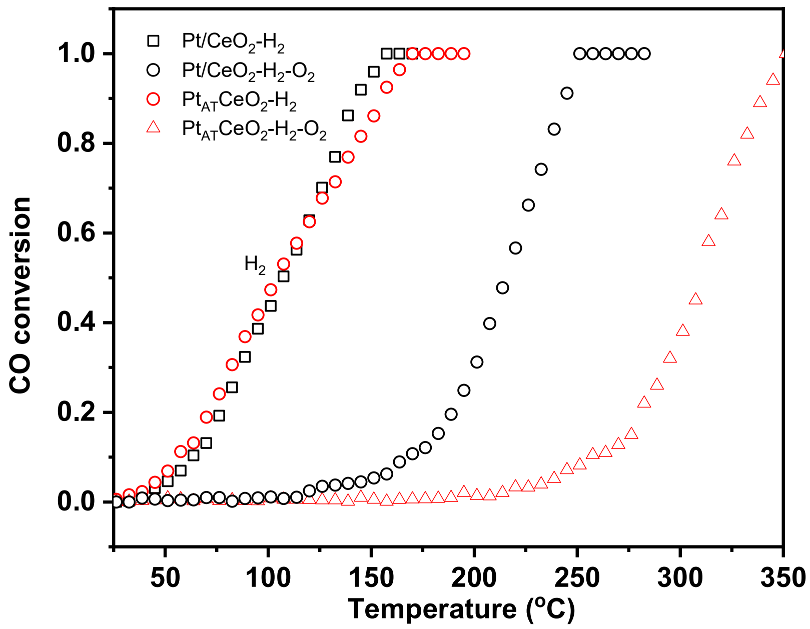

Fig. S30 CO oxidation performance (light-off curve) of Pt/CeO_2-H_2, $Pt_{AT}CeO_2$-H_2, Pt/CeO_2-H_2-O_2, $Pt_{AT}CeO_2$-H_2-O_2. Reaction conditions: catalyst loading = 20 mg, diliuted with SiC to 400 mg, 1% CO and 4% O_2 balanced in Ar, total flow rate = 100 mL/min, ramping from 20 to 500 °C with the ramping rate of 3 °C/min for each cycle

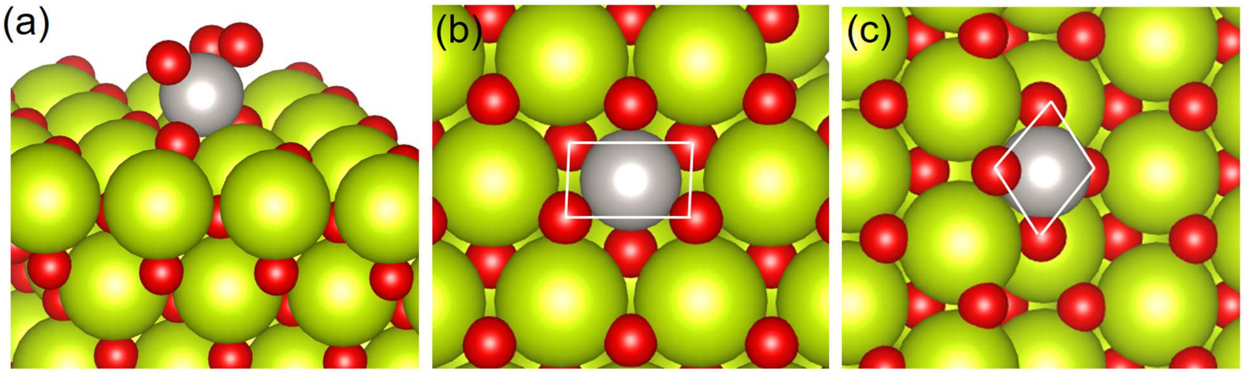

(d) EXAFS fits vs. DFT model (First shell Pt-O)

	XAFS Fit	DFT
**Pt/CeO$_2$**		
$N_{Pt–O}$	5.00 ± 0.43	5
$R_{Pt–O}$	2.00 ± 0.01	1.9-2.1
$\sigma^2 \times 10^3$ (Å^2)	2 ± 1	
ΔE_0 (eV)	4.48 ± 1.14	
R factor	0.003	
**Pt$_{AT}$CeO$_2$**		
$N_{Pt–O}$	4.89 ± 0.52	6 (surface)/4 (step)
$R_{Pt–O}$	2.00 ± 0.01	2.0-2.1 (surface)/1.9-2.0 (step)
$\sigma^2 \times 10^3$ (Å^2)	2 ± 1	
ΔE_0 (eV)	3.74 ± 1.45	
R factor	0.005	

Fig. S31 The optimized models of (a) adsorbed Pt_1, (b) square planar Pt_1 on CeO_2 surface and (c) square planar Pt_1 on CeO_2 step site, as well as the (d) comparison of EXAFS fits and DFT model with first shell Pt-O. Pt: grey, Ce: yellow, O: red

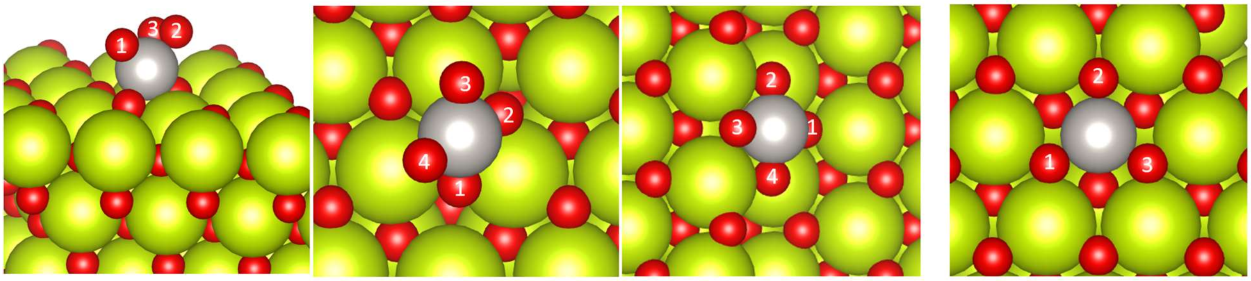

Sample	O vacancy formation energy (eV)			
	1	2	3	4
Adsorbed PtO_5	0.02	0.01	-0.25	~
Adsorbed PtO_4	0.73	0.28	0.23	0.41
Square planar Pt_1 on step	2.68	1.82	2.08	1.65
Square planar Pt_1on surface	1.14	1.15	1.12	~

Fig. S32 Oxygen vacancy formation energy of adsorbed Pt_1 and square planar Pt_1 on CeO_2(111) step and surface

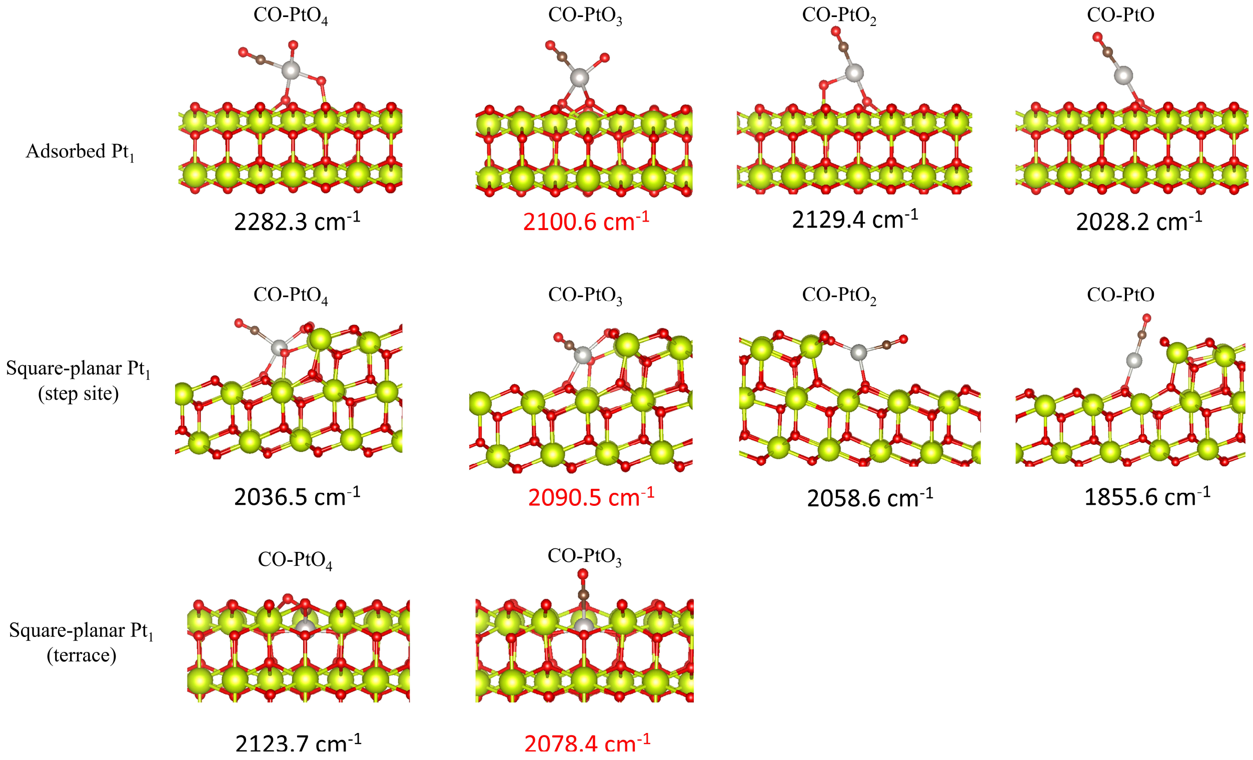

Fig. S33 Vibrational frequency of adsorbed CO on various PtO_x structures

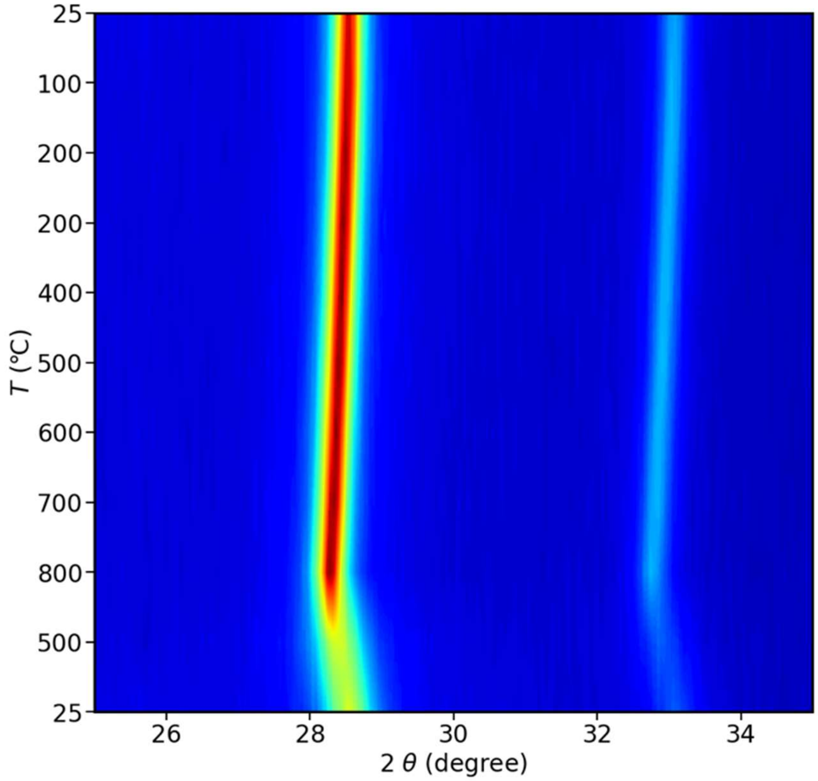

Fig. S34 *In situ* XRD intensity map for Pt/CeO_2 as a function of 2θ and temperature with the temperature first increases from 25 to 800 °C, and then decreases from 800 to 25 °C

2θ value decreases with the increase of temperature, and then increases as decreasing the temperature. This suggests the lattice expansion of CeO_2 during the ramping process, and lattice shrink during the cooling process. In other words, Ce-O distance in CeO_2 unit cell will be elongated at high temperature, which might be the driving force for the formation of square planar Pt_1. Also, the color represents the intensity of XRD pattern, in which red means higher intensity.

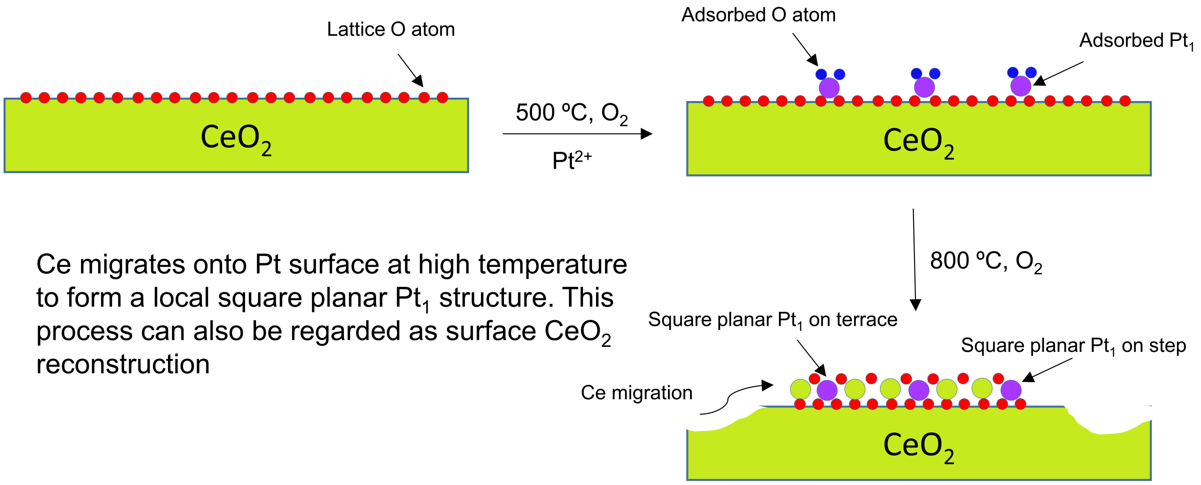

Fig. S35 The formation mechanism of adsorbed Pt_1 at low calcination temperature and square planar Pt_1 at high calcination temperature

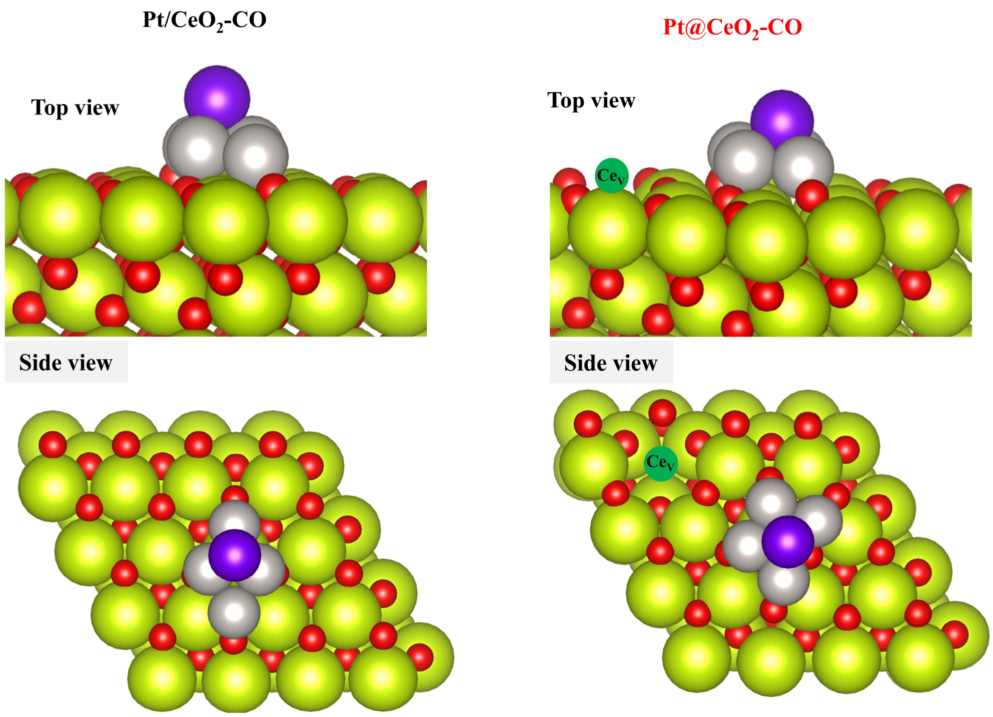

Fig. S36 The optimized models of Pt NPs (five Pt atoms) on CeO_2 (111) surface without Ce_v (left, Pt/CeO_2-CO) and with Ce_v (right, $Pt_{AT}CeO_2$-CO). Pt: grey, Ce: yellow, O: red. We mark top Pt atom as purple since the redispersion progress of this atom will be simulated later

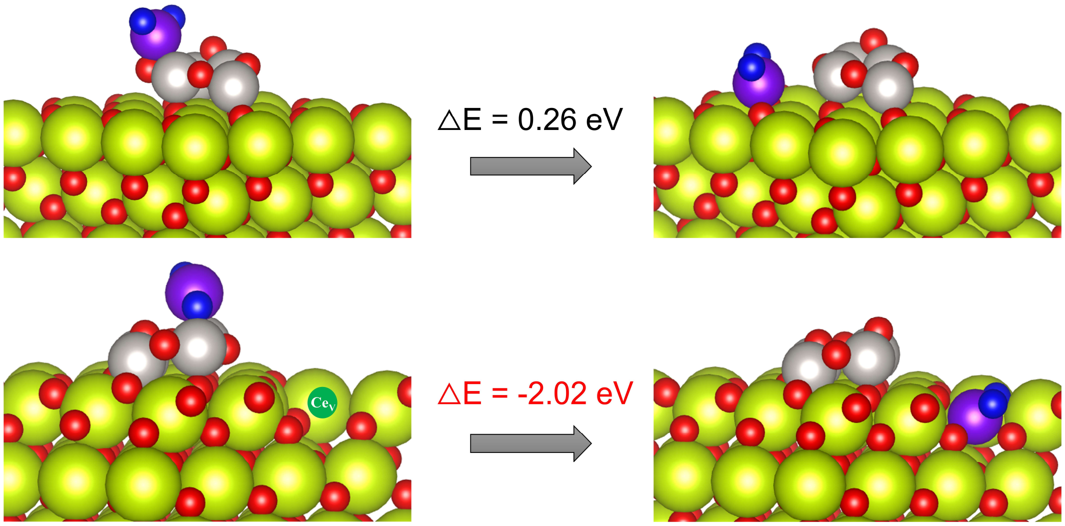

Fig. 37 The energy for redispersion of top Pt atom in PtO_x cluster to CeO_2 (111) surface and V_{Ce}. The energy is given in eV. Pt: grey, Ce: yellow, O: red, top Pt atom: purple, top O: blue
